# Whole systems approaches to obesity and other complex public health challenges: a systematic review

**DOI:** 10.1186/s12889-018-6274-z

**Published:** 2019-01-03

**Authors:** Anne-Marie Bagnall, Duncan Radley, Rebecca Jones, Paul Gately, James Nobles, Margie Van Dijk, Jamie Blackshaw, Sam Montel, Pinki Sahota

**Affiliations:** 1 Centre for Health Promotion Research, School of Health & Community Studies, Leeds Beckett University, Portland 519, Leeds, LS1 3HE UK; 2 School of Sport, Leeds Beckett University, Leeds, UK; 3 School of Clinical & Applied Sciences, Leeds Beckett University, Leeds, UK; 4 Diet & Obesity, Health Improvement Directorate, Public Health England, London, UK; 5 The National Institute for Health Research Collaboration for Leadership in Applied Health Research and Care West (NIHR CLAHRC West), University Hospitals Bristol NHS Foundation Trust, Bristol, UK; 6 Population Health Sciences, Bristol Medical School, University of Bristol, Bristol, UK

**Keywords:** Whole systems approaches, Obesity, Systematic review, Public health, Complexity, Systems science

## Abstract

**Background:**

Increasing awareness of the complexity of public health problems, including obesity, has led to growing interest in whole systems approaches (WSAs), defined as those that consider the multifactorial drivers of overweight and obesity, involve transformative co-ordinated action across a broad range of disciplines and stakeholders, operate across all levels of governance and throughout the life course. This paper reports a systematic review of WSAs targeting obesity and other complex public health and societal issues, such as healthy lifestyles for prevention of non-communicable disease.

**Methods:**

Seven electronic databases were searched from 1995 to 2018. Studies were included if there had been an effort to implement a WSA. Study selection was conducted by one reviewer with a random 20% double checked. Data extraction and validity assessment were undertaken by one reviewer and checked by a second reviewer. Narrative synthesis was undertaken.

**Results:**

Sixty-five articles were included; 33 about obesity. Most examined multicomponent community approaches, and there was substantial clinical and methodological heterogeneity. Nevertheless, a range of positive health outcomes were reported, with some evidence of whole systems thinking. Positive effects were seen on health behaviours, body mass index (BMI), parental and community awareness, community capacity building, nutrition and physical activity environments, underage drinking behaviour and health, safety and wellbeing of community members, self-efficacy, smoking and tobacco-related disease outcomes.

Features of successful approaches reported in process evaluations included: full engagement of relevant partners and community; time to build relationships, trust and capacity; good governance; embedding within a broader policy context; local evaluation; finance.

**Conclusions:**

Systems approaches to tackle obesity can have some benefit, but evidence of how to operationalise a WSA to address public health problems is still in its infancy. Future research should: (a) develop an agreed definition of a WSA in relation to obesity, (b) look across multiple sectors to ensure consistency of language and definition, (c) include detailed descriptions of the approaches, and (d) include process and economic evaluations.

**Electronic supplementary material:**

The online version of this article (10.1186/s12889-018-6274-z) contains supplementary material, which is available to authorized users.

## Background

In recent years, in response to the increasing awareness of the complexity of many public health problems, there has been growing interest in the role of systems-based approaches in public health. In 2007, the UK Foresight map [1] presented a pioneering portrayal of the complex web of obesity causation. In the same year the American Journal of Community Psychology devoted an edition to systems-thinking. In 2008 Mabry et al. [2] outlined the strategic vision of the Office of Behavioural and Social Sciences Research at the National Institutes of Health, listing systems science as one of four key programmatic directions, and the importance of systems thinking was also noted in the 2011 and 2015 Lancet Series on Obesity [3, 4].

In 2010 a number of evidence reviews were undertaken for the National Institute for Health and Care Excellence (NICE) [5–7], intended to inform the development of NICE guidelines on the prevention of obesity using a whole system approach (WSA) (note that the scope of the work was changed and instead resulted in the development of NICE guidelines on whole of community approaches) [5, 8].

The Garside et al. [5] review - one of those commissioned by NICE – which aimed to identify key elements of a WSA to obesity, reported that an “authentic” WSA draws on complexity science and complex adaptive systems. Although there is no consensus on a formal definition of “complex adaptive systems”, there is broad acceptance they contain: heterogeneous interacting elements; an emergent effect that is different from the effects of the individual elements; and persisting effects over time that adapt to changing circumstances [9]. The NICE reviews [5–7] did not find any “authentic” WSAs, and the definition was therefore widened to include those programmes that were designed to work at multiple levels among multiple agencies in a locality. Using this definition, they identified ten features of a WSA to tackle obesity [5] (Table 1).

**Table 1:** 10 features of a systems approach to tackle public health problems, adapted from NICE [9] and Garside et al. [5]

*Identifying a system.*	Explicit recognition of the public health system with the interacting, self-regulating and evolving elements of a complex adaptive system. Recognition given that a wide range of bodies with no overt interest or objectives referring to public health may have a role in the system and therefore that the boundaries of the system may be broad.
*Capacity building*	An explicit goal to support communities and organisations within the system.
*Creativity and innovation*	Mechanisms to support and encourage local creativity and/ or innovation to address public health and social problems.
*Relationships*	Methods of working and specific activities to develop and maintain effective relationships within and between organisations.
*Engagement*	Clear methods to enhance the ability of people, organisations and sectors to engage community members in programme development and delivery.
*Communication*	Mechanisms to support communication between actors and organisations within the system.
*Embedded action and policies*	Practices explicitly set out for public health and social improvement within organisations within the system.
*Robust and sustainable*	Clear strategies to resource existing and new projects and staff.
*Facilitative leadership*	Strong strategic support and appropriate resourcing developed at all levels.
*Monitoring and evaluation*	Well-articulated methods to provide ongoing feedback into the system, to drive change to enhance effectiveness and acceptability.

### Aims & objectives

The aim was to undertake a systematic review of national and international published evidence on WSAs targeting obesity, other public health areas and areas outside public health (such as social care, crime and justice), to understand what is known about WSAs and how they can be implemented in practice.

### Review questions

1. What has been done in terms of a WSA to obesity, and other complex public health problems, and how effective have these been?
2. What elements of a WSA are effective or not effective in (a) obesity (b) other areas of public health (c) areas other than public health?
3. What are the barriers and facilitators to implementing a WSA in (a) obesity (b) other areas of public health (c) areas other than public health?
4. What is the evidence on cost-effectiveness of WSAs in (a) obesity (b) other areas of public health (c) areas other than public health?

## Methods

This systematic review follows standard methodological guidelines [10, 11].

### Search strategy

A broad, sensitive search strategy was designed. The following databases were searched from January 1995 to September 2015 using a combination of text and Medical Subject Headings (MeSH terms): MEDLINE, CINAHL, Social Science Citation Index, The Cochrane Library (includes CENTRAL, DARE, NHSEED, HTA and INAHTA databases), PsycLIT/ PsycINFO, DoPHER, TRoPHI and IDOX.

In February 2017 an additional update search was run in MEDLINE, using the same search strategy applied from January 2015 to February 2018.

Key search terms included:

(i) “whole systems approach” and related terms such as holistic; cross-sector; systems-based approach; multi-strategy approaches etc.

OR

(ii) Terms related to relevant initiatives such as: Healthy Cities; Healthy Towns; Together Let’s Prevent Childhood Obesity (EPODE); Change4Life etc.

We also manually searched the websites of relevant organisations such as: Department of Health; Public Health England (PHE); Local Government Association (LGA); World Health Organisation (WHO); National Institute for Health and Care Excellence (NICE); Association for the Study of Obesity (ASO); National Obesity Forum etc.

The full search strategy is available as Additional file 1.

### Study selection

Titles and abstracts from electronic database searches were transferred to EPPI-Reviewer 4 [12], and screened against the inclusion criteria (Table 2). A random 20% of titles and abstracts were screened by all the review team, and once good agreement (80% or more) was reached, the remaining 80% were allocated to a single reviewer. Any queries were discussed within the review team and if agreement could not be reached, were referred to the local steering group for decisions. Records which potentially met the inclusion criteria, including those found on organisational websites, were retrieved in full and assessed for inclusion.

**Table 2:** Inclusion criteria

Inclusion criteria	Include	Exclude
Population	Any population where a WSA has been used, at local, regional, national and international level	
Intervention	WSAs, defined as those that: • Consider, in concert, the multifactorial drivers of overweight and obesity, as outlined by Foresight [1], public health or the social determinants of health [13]; • Involve transformative co-ordinated action (including policies, strategies, practices) across a broad range of disciplines and stakeholders, including partners outside traditional health sectors; • Operate across all levels of governance, including the local level so that such approaches are reinforced and sustained, and • Identify and target opportunities throughout the life course (from infancy to old age)	• Multiagency partnership working across sectors e.g. health & social care, but not at more than one level; case management initiatives focused on individuals or individual families; • Studies which looked at only one part of a WSA *(*i.e. one specific intervention delivered as part of a wider approach).
Comparator interventions	Any or none	
Outcomes	*Review questions 1 and 2*: • Health outcomes, e.g. weight, Body Mass Index (BMI), type 2 diabetes, diet and nutrition, physical activity, psychological well-being & quality of life; co-morbidities related to obesity, reductions in health inequalities, reductions in premature morbidity and mortality, cardiovascular disease and obesity-related cancers. • Organisational outcomes e.g. cross-sector collaboration; new partnerships; environmental changes; resource allocation; leadership etc. • Process outcomes, e.g. what each project aimed to achieve and barriers and facilitating factors associated with achieving or not achieving those aims. Outcomes may be at individual, local, regional or national/ federal/ principality level.	
	*Review question 3* Process and implementation outcomes e.g. training, recruitment, sustainability, people’s views on barriers and facilitators to implementation of WSAs.	
	*Review question 4*: Cost, cost-effectiveness, cost-benefit or cost-utility.	
Study designs	*Review questions 1 and 2*: primary research or evaluation studies. These may be randomised controlled trials (RCTs) or non-RCTs, natural experiments, before and after studies, or mixed methods evaluations (including case study approaches). *Review question 3*: process evaluations (qualitative or mixed method studies). *Review question 4*: cost-effectiveness, cost-benefit or cost-utility studies.	

### Data extraction

Data were extracted from included articles by one reviewer into a piloted electronic form, and checked by the lead reviewer. Queries were resolved as above. We extracted data into the following fields: study details; study design; setting; population (including PROGRESS-Plus indicators [14]); public health or other issue; intervention; comparator (if appropriate); outcomes; findings; reviewer comments.

We also assessed all included studies against the ten features for WSAs for obesity, in the working definition prepared by Garside et al. for the NICE guidance [5].

### Validity assessment

Two reviewers carried out validity assessment of included articles using checklists developed for quantitative and qualitative study designs of public health interventions [15]. These were adapted from the National Institute for Health and Care Excellence Public Health methods guidance, and the Critical Skills Appraisal Programme [16, 17]. Studies were given a quality rating based on how many criteria they met on the appropriate checklist.

### Synthesis

Due to the substantial clinical and methodological heterogeneity of included studies, a narrative approach to synthesis was chosen [18]. Evidence on health and non-health outcomes is presented as a descriptive thematic summary, grouped within each review question according to whether it relates to obesity, other public health or non-health issues, with the most methodologically robust evidence presented first.

We also looked at whether there was any association or pattern between the direction of reported health effects and the number of WSA features [5] that a study met, using the cross-tabulation function in EPPI-reviewer and carrying out a Fisher’s exact test in IBM SPSS 24 statistical software.

Qualitative evidence on barriers and facilitators to implementation and delivery of WSAs was summarised using a framework synthesis approach, allowing themes to emerge inductively from the included studies, within the framework of ‘barriers’ and ‘facilitators’. The framework was agreed within the review team, and data were aggregated according to the major themes.

## Results

### Study selection process

**Fig. 1:**
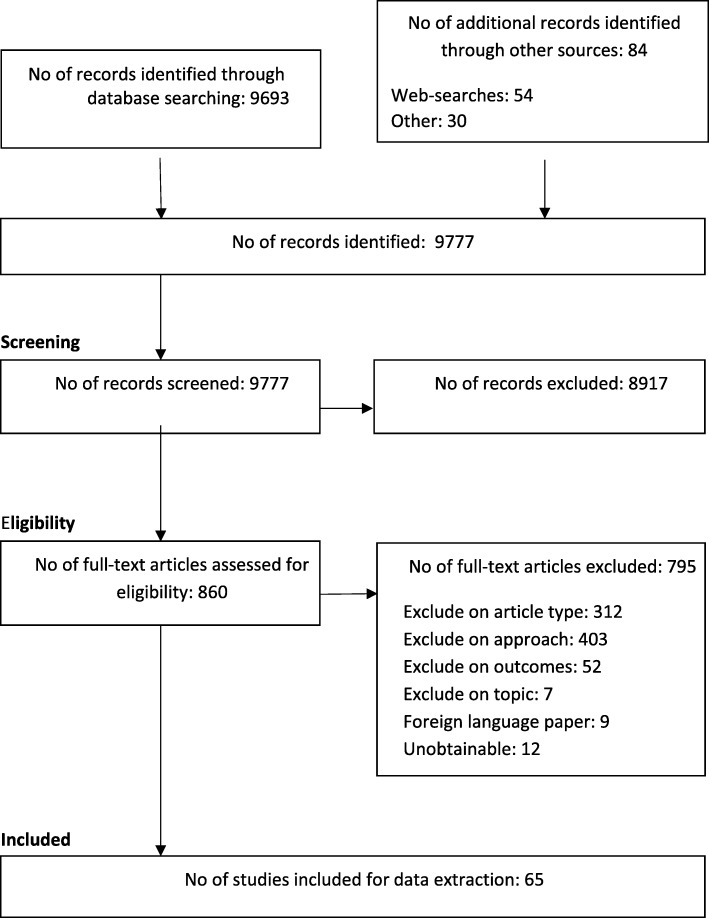
Study selection flow chart

Nine thousand seven hundred seventy-seven records were screened at title and abstract stage, 860 were retrieved in full and a total of 65 articles were included in the review [19–83]. Figure 1 depicts the study selection process and Additional file 2: Table S1 presents key characteristics of the included studies.

### Description of included studies

Table 3 gives an overview of public health issues addressed by study design and country that the research was carried out in. It is important to note that some studies sought to address more than one issue and many of the included studies were assessed as having more than one study design, for example Copeland et al. [25] combined mixed method evaluation techniques with a case control study nested within a prospective cohort study. The majority of the evidence was from the USA and Canada, though a substantial proportion also came from the UK, and from the Global WHO Healthy Cities network. Obesity and healthy lifestyle promotion were the dominant public health issues tackled, along with alcohol, smoking and drugs.

**Table 3:** Public health issue addressed in studies, by country

	Obesity	Healthy lifestyles	Smoking	Alcohol	Drugs	Diabetes	Cardiovascular disease	Falls prevention	Healthy weight gain in pregnancy	Chronic disease management	Other (domestic or alcohol related violence or disorder)
USA & Canada	TOTAL = 12 6 MME [19, 26, 37, 68, 75, 82] 1 survey [34] 1 nRCT [35] 1 Qual [76] 1 CS [42] 1 NAS [72]	TOTAL = 8 2 Qual [46, 74] 1 P cohort [23] 2 MME [45, 47] 2 nRCT [43, 57] 1 B&A [66]	TOTAL = 7 2 MME [49, 50] 2 CS [51, 56] 1 RCT [54] 1 nRCT [60] 1 B&A [63]	TOTAL = 2 2 RCT [48, 80] 1 MME [80] 1 Qual [80]		TOTAL = 2 1 MME & Qual [68] 1 Qual [74]	1 nRCT [43]		1 MME [37]	1 MME [45]	
UK	TOTAL = 7 1 MME [32] 1 survey [53] 4 Qual [38, 58, 61, 71] 1 B&A [53]	TOTAL = 3 2 Qual [24, 65] 1 MME [25]		1 MME/ B&A [55]	1 nRCT [79]			1 Qual [24]			1 MME/ B&A [55]
Australia	TOTAL = 5 1 MME [27] 2 Nat exp. [31, 32] 2 nRCT [44, 69]										
Iran		2 nRCT [59, 70]									
Netherlands		1 P cohort [73]					1 P cohort [73]				
Sweden		1 Qual [20]		1 Qual [78]							
WHO Healthy Cities (Global)		TOTAL = 9 8 MME [28, 29, 36, 40, 41, 52, 67, 77] 1 Qual [39]									
WHO Healthy Cities (Spain)		2 Qual [21, 22]									
WHO Healthy Cities (Germany)		1 MME [64]									
WHO Healthy Cities (Israel)		1 Survey [33]									
Who Healthy Cities (Bangladesh)		1 Qual [81]									
Other Global	2 Qual [62, 83]										

*MME* Mixed methods evaluation, *RCT* Randomised controlled trial, *nRCT* Non randomised controlled trial, *Qual* Qualitative or case study, *CS* Cross-sectional study, *NAS* Network analysis study, *B&A* Before and after study, *Nat Exp* Natural experiment, *P cohort* Prospective cohort study

#### Population

Forty-three studies included adults [19–22, 26, 32–36, 38, 39, 43–50, 52, 54–56, 58–66, 69–74, 78, 80, 81, 83] and 51 included children [19–23, 25–27, 30–32, 34–36, 38–40, 42–48, 50–53, 55, 56, 58–65, 68–73, 76, 78–83]. Fourteen focused on areas of socioeconomic deprivation [23, 44, 48, 50, 58, 61, 66, 68, 69, 73–76, 83] and ten on people in black or minority ethnic groups [23, 40, 49, 50, 56, 57, 66, 74, 76, 83]. Nine studies specified that they included older people [20, 22, 24, 34, 40, 45, 61, 66, 71]. Four studies targeted socially excluded groups [24, 66, 74, 75], two looked at women only [37, 74], two targeted people with disabilities [24, 74] and one targeted a specific religious or cultural group [45].

### Results of validity assessment

#### Quantitative studies

Methodological details were on the whole poorly reported. Twenty of the 44 quantitative studies did not have a comparator group. The comparator group was judged to be appropriate in 14 of the remaining 24 studies, and in 10 studies there was judged to be baseline equivalence between the groups. Eleven studies reported randomised assignment of participants to groups. Fourteen studies allocated interventions at the right level (e.g. cluster randomisation for school-level interventions); ten studies did not report sufficient information to judge allocation. Intention to treat analysis was reported in two of the three RCTs. The intervention was considered to have been described adequately in almost all the studies (*n* = 39). Unbiased intervention delivery (i.e. intervention and comparator groups treated the same, apart from the intervention) was reported in 20 of the 44 studies. The sample was judged to be representative of the population in 17 of the included studies, while in 28 studies the sample size was judged to be large enough. Potential confounding factors were adjusted for in 11 studies. Due to the nature of the interventions, blinded assignment to groups did not occur in any of the included studies, nor was blinded outcome assessment reported. Nine studies reported on attrition rates. In 29 studies, the analysis methods were judged to be appropriate, and a further 14 studies did not provide enough detail about the methods of analysis for the review team to assess whether they were appropriate.

#### Qualitative studies

Of the 30 studies with a qualitative design, the design was judged to be appropriate in 28 studies, with the remaining two providing insufficient detail. Seventeen studies provided a clear statement of findings, six provided insufficient detail, and the remaining seven did not provide a clear statement of findings. Fifteen studies used appropriate strategies for data collection (and 13 did not provide enough information), and 13 recruited participants appropriately (with 16 not providing enough information). Seven studies undertook rigorous data analysis, with three failing on this criterion and the remaining 20 providing insufficient information. Only three studies showed evidence of reflexivity regarding the relationship of the researcher to the participants, 18 failed on this criterion and the remaining 9 did not provide enough information. Nine studies provided enough information to satisfy the review team that ethical issues had been considered, while 21 did not provide enough information.

### Review question 1: What has been done in terms of a WSA to obesity, and other complex public health problems, and how effective have these been?

#### Studies meeting all ten guidance features for WSA

Thirteen included studies met all ten of the Garside et al. [5] features for a WSA [25, 29, 39, 46, 47, 53, 64, 65, 67, 75–77, 80]. Five of these 13 studies reported health or wellbeing outcomes [25, 29, 53, 75, 80], two reported outcomes associated with the social determinants of health [29, 80], and eleven reported process outcomes [25, 29, 39, 46, 47, 64, 65, 75–77, 80]. Three of these were UK initiatives, which all aimed to address obesity and healthy lifestyle promotion: a poor to moderate mixed methods evaluation of Change 4 Life [25], which reported positive outcomes for social determinants of health and mixed outcomes for health and wellbeing; a poor quality before and after study of Health Heroes [53], which reported positive health and wellbeing outcomes, and a poor quality case study of Public Health England’s paths to public health and wellbeing [65], which reported on process outcomes. Of the remaining 10 articles, seven were about the WHO Healthy Cities initiative [29, 39, 46, 47, 64, 67, 77] and all reported on process outcomes only. One of these was of poor to moderate quality [47], one was of moderate to good quality [39], one was good quality [46],and the remaining four were poor quality [29, 64, 67, 77]. The remaining three articles were: a poor quality mixed methods evaluation of the Central California Regional Obesity Prevention Program [75], which reported positive health outcomes; a poor quality case study of the San Diego Healthy Weight Collaborative [76], which reported process outcomes only, and a moderate to good quality randomised controlled trial of “Communities mobilizing for change on alcohol” [80], which also reported positive health outcomes.

#### All included studies

The following sections briefly summarise the findings of the included studies, with evidence of the highest methodological quality presented first. For further details, refer to Additional file 2: Table S1.

- Obesity

Positive effects on BMI and health behaviour were reported in two good quality non-RCTs of **Be Active Eat Well (BAEW)** in Australia, which met 7 out of the 10 WSA features [44, 69]; on BMI in a moderate to good quality non-RCT of **“Shape up Somerville”** in the USA [35], which met 8 out of the 10 NICE guidance features; on parental health behaviour & awareness in a mixed methods evaluation of moderate quality of the first year of the UK social marketing initiative **Change 4 Life** (C4L) [32], which met 8 out of the 10 WSA guidance features; on fitness and obesity in a moderate quality prospective cohort study of **Healthy Living Cambridge Kids** [23] in the USA, which met 4 of the 10 WSA guidance features; on BMI, parental awareness and community capacity building in two moderate to poor quality mixed methods evaluations of **Romp and Chomp** in Australia, which met 9 out of the 10 WSA guidance features [27, 31]; and on nutrition and physical activity environments in an evaluation (of unclear design or quality) of the **Central California Regional Obesity Prevention Program** (CCROPP) [75], which met all 10 WSA guidance features.

Mixed effects on health and wellbeing outcomes, with no reduction in obesity prevalence, were seen in a moderate to poor quality nested case control study of “Healthy Towns” in the UK [25], which met all 10 of the WSA guidance features; and mixed effects were reported for obesity prevalence in a moderate quality non-randomised trial of **Travis County CATCH** in the USA [42], which met none of the 10 WSA guidance features.

A poor quality mixed methods evaluation of LIVE 5–2–1-0 reported early indications of improvements in community awareness and action to promote healthy childhood behaviours, from stakeholder interviews.

(b) Other public health issues

Positive effects on underage drinking behaviour and health, safety and wellbeing of community members were reported in a moderate to good quality RCT of the USA initiative **Communities Mobilising for Change on Alcohol (CMCA)**, which met all 10 WSA guidance features [80]; on self-efficacy and intentions in a moderate quality non-RCT of a **community-based intervention** in Indigenous populations in the Canadian Arctic [57], which met 9 out of the 10 WSA guidance features; in smoking and tobacco-related disease outcomes in a cross-sectional survey of moderate quality, and a before and after study of poor quality, of the **California Tobacco Control Program (CTCP)** [56, 63], which met 2 out of the 10 WSA guidance features; on smoking prevalence in a prospective cohort study of moderate to poor quality [50] of the **Racial and Ethnic Approaches to Community Health (REACH)** project, in the USA, which met 8 out of the 10 NICE guidance features; in healthy eating behaviour in a non-RCT of moderate to poor quality of The **Isfahan Healthy Heart programme** evaluation in Iran [59], that met 6 out of 10 WSA guidance features; on smoking rates, home smoking bans and support for smoke-free policies in a repeated cross-sectional survey of poor quality of **Put it Out Rockland (PIOR)** in the USA [51], which met 3 out of the 10 WSA guidance features; and on access to health and medical services for managing diabetes, and access to fresh food in a poor quality case study on **community-based participatory diabetes prevention** [74], which met only one of the 10 WSA guidance features.

Mixed health effects were reported on risk factors for cardiovascular disease in a moderate quality prospective cohort study of a community intervention in the Netherlands [73], which met 7 of the 10 WSA guidance features; and on BMI in a moderate to poor quality non-RCT [43] of the Minnesota Heart Health Programme (MHHP), which met 4 of the 10 WSA guidance features.

### Review question 2: What elements of a WSA are effective or not effective in (a) obesity (b) other areas of public health (c) areas other than public health?

**Fig. 2:**
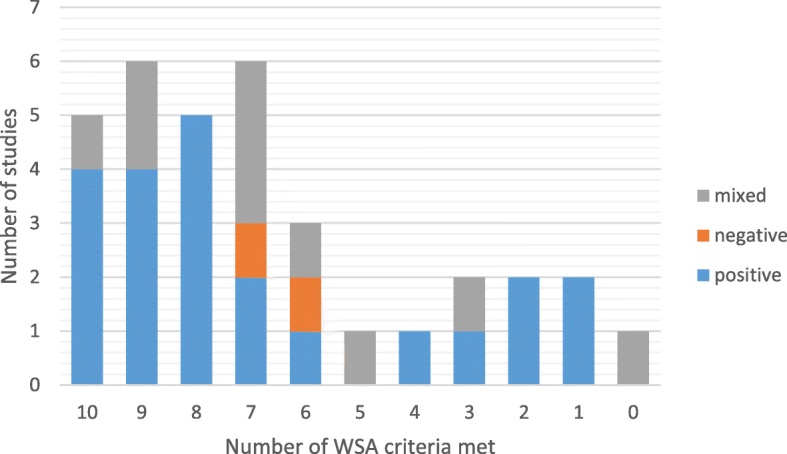
Total number of WSA features met versus direction of health effects

**Fig. 3:**
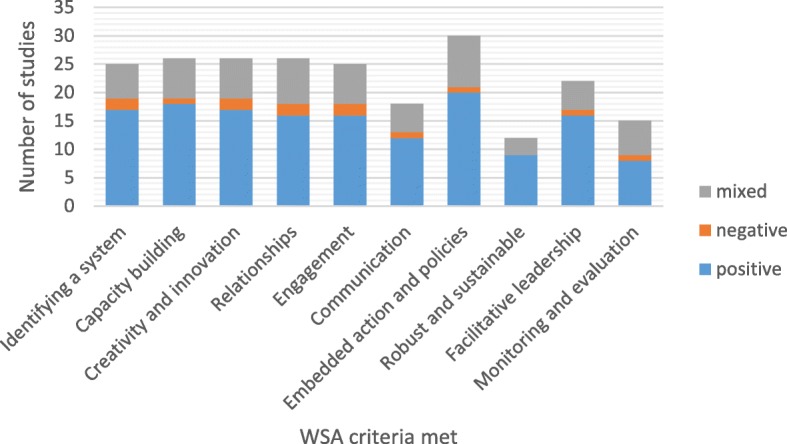
Individual WSA features met versus direction of health effects

Studies of interventions which met a large number (8–10) of the WSA guidance features frequently reported positive health effects when looking at the descriptive statistics (see Fig. 2 and Table 4). The Fisher’s exact test determined that the association between WSA feature categories (0–3 features, 4–7 features, or 8–10 features) and the health effects was not statistically significant (df 4, chi-square value 6.645, *p* = 0.094). All of the 10 individual WSA guidance features were associated with positive health outcomes (see Fig. 3) but the overall association was not statistically significant (df 40, chi-square value 45.20, *p* = 1.000). These statistical findings should be interpreted with caution given the small number and heterogeneous nature of the studies, and that only studies that reported on health outcomes could be included in this analysis (34 out of a total of 65 studies included in this review). Furthermore, only five of the 13 studies that met all 10 WSA guidance features reported health outcomes; the remaining nine were process evaluations and therefore not included in this analysis.

**Table 4:** Number of WSA criteria met versus direction of health effects

Number of Criteria Met	Positive	Negative	Mixed
8–10 (Systems embedded)	13	0	3
4–7 (Systems moderately embedded)	4	2	5
0–3 (Systems not embedded)	5	0	2

Fisher’s exact test (df 4, chi-square value 6.645) *p* = 0.094

### Review question 3: What are the barriers and facilitators to implementing a WSA in (a) obesity (b) other areas of public health (c) areas other than public health?

Key themes that emerged from the included process evaluations were as follows (with evidence of the highest methodological quality which met 6 or more of the WSA features (e.g. Healthy Cities) presented first):

1. **Strong leadership and full engagement of all partners is key for success**: Senior leadership buy-in was important to the effective development and delivery of a number of initiatives [65, 77]. Evaluations of the Healthy Cities initiative [39, 22] reported that effective organisational change required core principles to be integrated into an organisation’s mainstream activity, and recognised the need to have a genuine consortium of partners who were actively engaged rather than a single main driving force.
2. **Engaging the local community is an important component of a successful approach**: Several evaluations showed that projects were successful because of effective community involvement in identifying their needs and actively participating in local solutions [47, 49–51, 57, 74, 75, 77, 80, 82]. Capacity building through coalitions, targeted actions and community and system change were also identified as important [49, 50].
3. **Creating successful outcomes requires time to build relationships, trust and community capacity**: Local authority action built up good working relationships based on mutual trust, shared vision, aims and values, which were of central importance to developing and delivering effective work. A Public Health England report [65] suggested that where such partnerships were not already in place, it was worth investing time and effort to develop them.
4. **Good governance and shared values**: Distinguishing features of the WHO European Healthy Cities Network are shared vision and values and an explicit commitment to good governance by local councils and their executive arms [29, 39]. The evaluations showed that the emphasis on good governance resulted in better participation, policy-making and intersectoral action. This happened across the designated cities and was not limited to a certain class (in terms of population or geographical location) [28].
5. **Appropriate partnerships are important to create sustainable multilevel environmental change:** Building an effective and sustainable collaborative team was found to be key [46, 65, 76, 83]. Identifying key stakeholders and securing their early participation was considered important, as was building an appropriate network of collaborators [22, 27, 32, 65, 82]. Strong relationships between stakeholders was identified as one of the factors influencing effectiveness [62], whilst engaging non-traditional partners can expand reach [51].
6. **Consistency in language used across organisations**: The creation and use of a common language can overcome the fact that most organisations have their own beliefs and structures, for example by using consistent and authorised messages and materials, based on insights gained through prior research in the target community [32].
7. **Embedding initiatives within a broader policy context**: It is important to ensure agendas focus on the principles of what the organisations wish to achieve, and to integrate or align initiatives so they are not seen as additional to mainstream activity [39, 51].
8. **Local evaluations**: A number of studies reported that local evaluations to inform effectiveness of local level interventions from the outset were important [25, 51, 62].
9. **Sufficient financial support and resources**: Access to relevant resources, including funding, was identified as important in several process evaluations, and lack of resource was identified as a barrier to successful implementation of WSAs [27, 39, 46, 51, 62, 64, 65, 82, 83].

### Review question 4: What is the evidence on cost-effectiveness of WSAs in (a) obesity (b) other areas of public health (c) areas other than public health?

We found very little evidence relating to cost effectiveness of a WSA. Only one study, a cross-sectional survey of moderate to poor quality of Put it Out Rockland [51], reported an apparent cost-benefit comparison, although the methods used were not clear: with an estimated return of $4 to $5 for every dollar spent on tobacco control, Rockland’s total $6 million investment between 2000 and 2010, added to the state’s investment, translated to a potential $24 million to $30 million savings in tobacco- related costs for Rockland County. However the cross-sectional study design means that conclusions about impact cannot be drawn.

## Discussion

Although 65 studies met the broad inclusion criteria, the heterogeneity of studies in terms of different outcomes, research designs, populations and interventions, prevented the data from being analysed statistically. It is also worth noting that most of the included studies did not set out to implement or evaluate a WSA. Furthermore, reporting of intervention and approaches in published articles was usually brief and lacked detail. This underlines the lack of robust evidence in this area and the need for further research to expand and support the suggested associations, in order to increase our understanding of how WSAs can be implemented. Nevertheless, it does seem reasonable that programmes in a community setting that adopt the principles of the ten features identified by Garside et al. [5] such as developing relationships and engaging stakeholders, ensuring the approach is robust and sustainable and having supportive leadership, are more likely to be successful than programmes that do not adopt these principles. Similarly, it is also feasible that all ten of the features [5] are associated with positive health effects, however in relation to current thinking around systems approaches, these ten features do not comprehensively describe a WSA.

The review also found consistent evidence from process evaluations that ownership and commitment, strong relationships between stakeholders, and allowing sufficient time to build relationships, trust and community capacity are all key to building a successful WSA*.*

There is recognition in the literature that several public health problems, including obesity, are complex issues requiring system-based approaches [84, 85]. However, although the concepts and terminology have existed for some decades the degree to which the field has progressed is debatable, partly due to the multitude of ways in which the language surrounding systems approaches is used.

Systems science “*refers to a range of methods, composed largely of mathematical or computational modelling and simulation, that enable the user to explore complex problems by addressing both interactions between components of a system and the behaviour of the system over time*” [2]. On the one hand, there have been significant advances in the application of a range of systems science approaches to a variety of obesity-related public health issues using techniques such as micro-simulation, social network analysis, agent-based modelling and system dynamics modelling. On the other hand, Garside et al. (2010) reported that the term “whole system approach” was found to represent approaches informed by theory about complex systems which propose new ways of organising, managing and evaluating activities, and also as terminology within a long list of approaches which referred to cross-disciplinary, multi-agency, multi-level community activities aimed at addressing health concerns affected by complex socio-economic conditions [5] and which rarely, if ever, encompass the use of the system science methods as described above.

Similarly to Garside et al. [5], this review found little evidence of systems science or systems thinking in included studies. Few programmes had been explicitly designed and delivered with an a priori recognition of the public health issue as a system and thus rarely approached implementation from a perspective encapsulating a systems approach – the implications of which are significant for the reporting and evaluation of interventions. Furthermore, because interventions to date have not been undertaken with a systems-thinking lens or set out to take a WSA at the outset, there has been little recognition of properties inherent in a complex system (e.g. nonlinear relationships, feedback loops, dynamic interacting elements) and little attention afforded to the reporting of the central underlying operational mechanisms (e.g. improving networks, developing a common agenda, developing relationships), as suggested by Allender et al. [86]. Hawe et al. [85] noted that although the majority of health promotion programmes claim to take an ecological approach, in reality this is realised as multiple interventions at multiple levels with “*little theory put forward about how these levels impact the unfolding of the intervention or how they affect intervention outcomes*”. Moreover, several authors have noted that implementation of a suite of activities across multiple-settings or multiple-levels is not necessarily the same as taking a “systems approach” [5, 85, 87]. Systems approaches focus on the context into which the intervention is introduced [85] and relate to intervening directly on the feedbacks, structures and goals of a system [87]. Considered in this light the evidence highlights the limited progress that has been made in the practical implementation and evaluation of WSAs to public health issues to date. What is needed a framework to incorporate the complexity of systems approaches into public health research, policy and practice [88].

Garside and colleagues in 2010 also looked for examples of a whole system in action, finding only eight articles on the effectiveness of community wide programmes displaying features of a WSA to prevent obesity, none of which were undertaken in the UK and all of which targeted children below 14 years of age [6]. Most findings favoured the interventions but improvements were found to be small and not always statistically significant. The inconclusive evidence relating to the 10 NICE guidance features in the present review may be because these emerged from a systematic review with the aim of providing a working definition of a WSA to obesity prevention, which did not find any “authentic” WSAs, and the definition was therefore widened to include those programmes that were designed to work at multiple levels among multiple agencies in a locality [5]. So, meeting all ten of these criteria still does not indicate an “authentic” WSA. In addition, although studies may have met many or all of the 10 NICE guidance features in their description of their proposed intervention, in studies which presented effectiveness outcomes there was little evidence of whether these interventions had been implemented with fidelity to the WSA framework.

### Limitations

Our search strategy was designed to look as widely as possible to minimise the risks of missing valuable material. Although 20% of titles and abstracts were double screened, screening of the remaining 80% was limited to a single reviewer, an “acceleration strategy” recommended for rapid reviews [89]. While this was a pragmatic necessity, it does potentially introduce bias and human error, which may have resulted in some relevant studies being missed.

Methodological details of included studies were, on the whole, poorly reported, which limits our confidence that the findings are not at significant risk of bias.

Only 11 of the included studies were UK-based, however their findings might be expected to be generalisable to the UK context and three of these [32, 53, 65] met all ten of the NICE criteria for a WSA.

Few studies targeted population groups known to be at higher risk of obesity and other public health issues, such as black and minority ethnic groups and people with low levels of education or low socioeconomic status. This limits the usefulness of the findings.

Thirteen of the 65 included studies were judged to meet all ten of the criteria for a WSA proposed by Garside et al. [5] in an earlier review. However, the heterogeneity of studies in terms of interventions, outcomes, research designs, and populations prevented the data from being analysed statistically.

### Conclusions

Using a broad lens, this systematic review aimed to obtain a greater insight on the effectiveness of WSAs and how they can be implemented in practice. Evidence exists to demonstrate promise with interventions working towards systems approaches. This was most clearly demonstrated through a suite of WHO Healthy Cities process evaluations and evidence from whole of community approaches. A range of positive health outcomes were reported, but there was little evidence of an association between specific WSA features and health impacts. Evidence of systems science and systems thinking was less clear, even in the most “joined up” approaches, similar to the findings of the series of reviews carried out for NICE in 2010.

### Recommendations

It is important to note that most of the included studies did not report that they set out to implement or evaluate a WSA, and reporting of interventions and approaches in published articles was usually brief and lacked detail. This underlines the lack of evidence in this area and the need for further research. Whilst several learnings on multi-level, community wide interventions have been obtained which are likely to be relevant to the implementation of a true WSA (e.g. evidence about barriers and facilitators to implementing such approaches), it is also evident that evidence of how to operationalise a whole systems approach to address public health problems is still in its infancy. We recommend that future researchers and policy makers develop consistency in language and an agreed definition of what a WSA should be in relation to obesity. Future research studies into the effectiveness of WSAs should look across sectors and should include detailed descriptions of interventions including approaches, and embedded process and economic evaluations, as recommended by existing guidance on developing and evaluating complex interventions [90, 91].

## Additional files

Additional file 1: Search strategy for electronic databases & list of websites searched. (DOCX 18 kb)

Additional file 2: **Table S1.** Included studies. (DOCX 116 kb)

## Abbreviations

ASO: Association for the Study of Obesity
BAEW: Be Active Eat Well
BMI: Body Mass Index
CATCH: Coordinated Approach To Child Health
CCROPP: Central California Regional Obesity Prevention Program
CINAHL: Cumulative Index to Nursing and Allied Health Literature
CMCA: Communities Mobilising for Change on Alcohol
CTCP: California Tobacco Control Program
DARE: Database of Abstracts of Reviews of Effects
DoPHER: Database of Promoting Health Effectiveness Reviews
EPODE: Ensemble Prévenons l’Obésité Des Enfants’ (Together Let’s Prevent Childhood Obesity)
HTA: Health Technology Assessment
INAHTA: International Network of Agencies for Health Technology Assessment
LGA: Local Government Association
MHHP: Minnesota Heart Health Programme
NHSEED: National Health Service Economic Evaluation Database
NICE: National Institute of Health & Care Excellence
PHE: Public Health England
PIOR: Put it Out Rockland
RCT: Randomised controlled trial
REACH: Racial and Ethnic Approaches to Community Health
TROPHI: Trials Register of Promoting Health Interventions
WHO: World Health Organisation
WSA: Whole systems approach
