# Supplementary material for: Whole systems approaches to obesity and other complex public health challenges: a systematic review
Source: BMC Public Health. 2019 Jan 3;19:8. doi: 10.1186/s12889-018-6274-z (PMC6318991; doi:10.1186/s12889-018-6274-z)
Supplement: Supplementary file 1 — Search strategy for electronic databases & list of websites searched. (DOCX 18 kb) [file 12889_2018_6274_MOESM1_ESM.docx]

**Search Strategy for Whole systems approaches to obesity and other complex public health challenges: a systematic review (Bagnall et al.)**

1. Obes* or overweight or fat* or “body weight” or “weight loss” or leptogenic or (health* adj/2 (weight* or eat* or choice* or adiposity) or (weight adj/2 (gain* or change* or retention* or loss*)) or poverty or “social exclusion” or depriv* or diabetes or “wicked problem*” or “wicked issue*” or “troubled famil*” or (drug* adj/2 (use* or abuse*))
2. “whole system” or “system* approach*” or (system adj/2 work*) or collaborative or “joined up” or holistic or “cross sector” or “multi disciplinary” or “inter disciplinary” or integrated or (local* adj/2 wide) or (local* adj/2 cross) or “multi faceted” or “multi agency” or “community wide” or “inter organisation* or network* or “full system” or coordinated or aligned or systematic or “city wide” or (region* adj/2 wide) or (region* adj/2 cross) or regional or combined or united or “health system” or “public health” or “state wide” or (complex adj/2 system*)
3. Approach* or strateg* or policy or policies or initiative* or scheme* or program* or intervention* or prevention or control
4. “Healthy Cities” or “Healthy Town*” or EPODE or Change4Life or “Healthy Weight Healthy Lives” or “National Support Team Childhood Obesity” or “Healthy School*” or “Healthy Place*” or “Manchester Experiment”

Medline Subject Headings:

1. (MH "Obesity") OR (MH "Pediatric Obesity") OR (MH "Obesity, Morbid") OR (MH "Overweight") OR (MH "Body Weight") OR (MH "Weight Loss")

**Web based searches**

**Websites**

- The Association of Directors of Public Health website ([www.adph.org.uk/](http://www.adph.org.uk/)) was searched on 21/08/2015 using the on-site search engine with the single search term: ‘obesity’.
- The Association for the Study of Obesity website ([www.aso.org.uk/](http://www.aso.org.uk/)) was searched on 21/08/2015.
- The British Heart Foundation National Centre for Physical Activity and Health website ([www.bhfactive.org.uk](http://www.bhfactive.org.uk)/) was searched on 21/08/2015 using the on-site search engine with the single search term: ‘obesity’. The section of the website labelled ‘research and evaluation’ was scanned in detail.
- The Core Cities website (www.corecities.com/) was searched on 21/08/2015 using the on-site search engine with single search terms: ‘obesity’ and ‘whole systems approach’.
- The Department of Health website ([www.gov.uk/government/organisations/department-of-health](http://www.gov.uk/government/organisations/department-of-health)) was searched using the on-site search engine with single search terms: ‘obesity’ and ‘whole system approach’.
- The Diabetes UK website ([www.diabetes.org.uk](http://www.diabetes.org.uk) ) was searched on 21/08/2015 using the on-site search engine with single search terms: ‘obesity’ and ‘whole systems approach’. The section on the website labelled ‘research’ was scanned in detail.
- The Faculty of Public Health website ([www.fph.org.uk/](http://www.fph.org.uk/)) was searched on 21/08/2015 using the on-site search engine with single search terms: ‘obesity’ and ‘whole systems approach’. The section on the website labelled ‘publications’ was scanned in detail.
- The Health Foundation website ([www.health.org.uk/](http://www.health.org.uk/)) was searched on 21/08/2015 using the on-site search engine with single search terms: ‘obesity’ and ‘whole systems approach’. The section on the website labelled ‘publications’ was scanned in detail.
- The King’s Fund website (<http://www.kingsfund.org.uk/>) was searched on 21/08/2015 using the on-site search engine with the single search term: ‘obesity’. The section of the website labelled ‘publications’ was scanned in detail.
- The LGA website ([www.local.gov.uk](http://www.local.gov.uk)/) was searched on 21/08/2015 using the on-site search engine with single search terms: ‘obesity’ and ‘whole systems approach’. The section on the website labelled ‘publications’ was scanned in detail.
- National Institute for Health and Care Excellence (NICE) website ([www.nice.org.uk/](http://www.nice.org.uk/)) was searched on 21/08/2015 using the on-site search engine with single search terms: ‘obesity’ and ‘whole systems approach’. The section on the website labelled ‘research’ was scanned in detail.
- The National Obesity Forum website ([www.nationalobesityforum.org.uk/](http://www.nationalobesityforum.org.uk/)) was searched on 21/08/15. The sections of the website labelled ‘British Journal of Obesity’ and ‘publications’ were scanned in detail.
- The New Economics Foundation website ([www.neweconomics.org/](http://www.neweconomics.org/)) was searched on 21/08/2015 using the on-site search engine with single search terms: ‘obesity’. The section on the website labelled ‘publications’ was scanned in detail.
- Obesity Learning Centre website ([www.ncdlinks.org/olc/](http://www.ncdlinks.org/olc/)) was searched on 21/08/2015.
- The Public Health England website ([www.gov.uk/government/organisations/public-health-england](http://www.gov.uk/government/organisations/public-health-england)) was searched on 21/08/2015 using the on-site search engine with single search terms: ‘obesity’ and ‘whole systems approach’.
- The Royal Town Planning Institute website ([www.rtpi.org.uk/](http://www.rtpi.org.uk/)) was searched on 21/08/2015.
- The SOLACE website ([www.solace.org.uk/](http://www.solace.org.uk/)) was searched on 21/08/2015. The sections on the website labelled ‘articles’ and ‘consultation processes’ were scanned in detail
- Town and Country Planning Association website ([www.tcpa.org.uk/](http://www.tcpa.org.uk/)) was searched on 21/08/2015 using the on-site search engine with single search term: ‘obesity’.
- The UK Public Health Association website ([www.ukpha.org.uk/](http://www.ukpha.org.uk/)) was searched on 21/08/2015.
- The World Health Organisation (WHO) website ([www.who.int/en/](http://www.who.int/en/)) was searched on 21/08/2015 using the on-site search engine with single search terms: ‘obesity’ and ‘whole systems approach’.
- The Scottish Government website ([www.scotland.gov.uk/Home](http://www.scotland.gov.uk/Home)) was searched on 09/09/2015. The sections on the website labelled ‘topic: health and social care: healthy living’ were scanned in detail.
- The NHS Health Scotland website ([www.healthscotland.com/](http://www.healthscotland.com/)) was searched on 09/09/2015 using the on-site publications search engine with the single search term: ‘obesity’.
- The NHS Health Scotland website ([www.healthscotland.com/](http://www.healthscotland.com/)) was searched on 10/09/2015 using the on-site publications search engine within the category ‘research reports’.
- The NHS Health Scotland website ([www.healthscotland.com/](http://www.healthscotland.com/)) was searched on 10/09/2015. The sections on the website labelled ‘health topics’ were scanned in detail.
- The Joseph Rowntree website ([www.jrf.org.uk/](http://www.jrf.org.uk/)) was searched on 10/09/15 using the onsite search engine with single search terms: ‘diet’ and obesity’.
- The National Institute for Clinical Excellence website ([www.nice.org.uk/](http://www.nice.org.uk/)) was searched on 14/09/15. The section on the website labelled ‘NICE guidance: public health guidelines’ was scanned in detail.
- The “this city’s going on a diet” website ([www.thiscityisgoingonadiet.com/](http://www.thiscityisgoingonadiet.com/)) was searched on 24/02/2016.
- The Wellcome Trust website ([www.wellcome.ac.uk/](http://www.wellcome.ac.uk/)) was searched on 29/02/2016.
- The Sorrell Foundation website ([www.thesorrellfoundation.com/](http://www.thesorrellfoundation.com/)) website was searched on 29/02/2016.
- The More Life website ([www.more-life.co.uk/](http://www.more-life.co.uk/)) was searched on 29/02/16. The section on the website labelled ‘obesity’ was scanned in detail.
- The National Institute for Health Research website ([www.nihr.ac.uk/](http://www.nihr.ac.uk/)) was searched on 29/02/2016 using the onsite search engine with single search terms: ‘obesity’ and ‘whole system’.
- The Nutrition Society website (<http://www.nutritionsociety.org/news>) was searched on 29/02/2016.
- The British Nutrition website (<https://www.nutrition.org.uk/>) was searched on 01/03/2016 using the on-site search engine with single search terms: ‘obesity’ and ‘whole system’.
- The York Health Economics Consortium (<http://www.yhec.co.uk/>) was searched on 01/03/2016.
- The Medical Research Council website (<http://www.mrc.ac.uk/>) was searched on 03/03/2016 using the on-site search engine with single search terms: ‘obesity’ and ‘whole system’. The section on the website ‘published research’ was scanned in detail.
- The Economic and Social Research Council website (<http://www.esrc.ac.uk/>) was searched on 03/03/2016 using the on-site search engine with single search terms: ‘obesity’ and ‘whole system’. The section on the website ‘publications’ was scanned in detail.
- The Big Lottery Fund website (<https://www.biglotteryfund.org.uk/>) was searched on 03/03/2016 using the on-site search engine with single search terms: ‘obesity’. The section on the website ‘research’ was scanned in detail.
- The Health Equalities Group website (<http://www.hegroup.org.uk/>) website was searched on 03/03/2016. The sections on the website labelled ‘research’ and ‘projects’ were scanned in detail.
- The Nuffield Trust website (<http://www.nuffieldtrust.org.uk>) was searched on 03/03/2016 using the on-site search engine with single search terms: ‘obesity’ and ‘whole system’.
- The Imperial College London website (<http://www.imperial.ac.uk>) was searched on 03/03/2016 using the on-site search engine with single search terms: ‘obesity’ and ‘whole system’.
- The National Obesity Observatory website (<http://www.noo.org.uk>) was searched on 03/03/2016. The section on the website ‘our publications’ was scanned in detail.
- The Centres for Disease Control and Prevention website (<http://www.cdc.gov>) was searched on 07/03/16 using the on-site search engine with single search terms: ‘obesity’ and ‘whole system’.
- The Healthy Kids Healthy Communities website (<http://www.healthykidshealthycommunities.org>) was searched on 07/03/2016.
- The Robert Wood Johnson Foundation website (<http://www.rwjf.org/>) was searched on 07/03/16 using the on-site search engine with single search terms: ‘obesity’ and ‘whole system’.
- The EPODE International Network website (<http://epode-international-network.com/>) was searched on 07/03/2016.

**Search Engines**

- The Google search engine was searched on 09/09/2015 with the single search term: ‘whole system approach.’
- The Google search engine was searched on 09/09/2015 with the single search term : ‘whole system approach health’.
- The Google Scholar search engine was searched on 09/09/2015 with the single search term: ‘whole system approach’.
- The Google search engine was searched on 14/09/2015 with the single search term: ‘whole system approach public health’.
