# Supplementary material for: Whole systems approaches to obesity and other complex public health challenges: a systematic review
Source: BMC Public Health. 2019 Jan 3;19:8. doi: 10.1186/s12889-018-6274-z (PMC6318991; doi:10.1186/s12889-018-6274-z)
Supplement: Supplementary file 2 — Table S1. Included studies. (DOCX 116 kb) [file 12889_2018_6274_MOESM2_ESM.docx]

Additional file 2: **Table S1**: Included Studies

KEY: + positive; - negative; m mixed; ? unclear; SDH social determinants of health

| **Study** | **Public Health Issue** | **Study Design/ Methods** | **Population/Setting** | **Intervention** | **Outcomes** | **Quality** | **Garside et al. WSA criteria met** |
| --- | --- | --- | --- | --- | --- | --- | --- |
| Active Living by Design. (2014)(1) | Obesity  Childhood obesity. | Mixed methods evaluation.  Collaborative, community-based approach utilising multiple methods, site-specific assessment strategies and cross-site evaluation components. | Adults; Children/ Young People.  USA.  54% urban, 25% rural. | ***Healthy Kids, Healthy Communities:*** 49 communities, intentionally diverse in nature, proposed a range of healthy eating and active living strategies *e.g.* increasing community gardens and farmers’ markets, enhancing conditions for safe bicycling and walking, improving access to parks and playgrounds. | Process | Qual 1  Quant 1  POOR | 9 |
| Amed S, Shea S, Pinkney S, Wharf Higgins J, Naylor PJ (2016) (2) | Childhood obesity | Mixed-methods evaluation.  Surveys and stakeholder interviews. | Four communities in British Columbia, Canada: 2 large cities, one small city and one comprised of several small municipalities | ***Live 5-2-1-0*** – a multi-sector multi-component childhood obesity prevention initiative informed by systems thinking and participatory research via an innovative knowledge transfer model. | Process;  Community wellbeing + | Qual 5  Quant 7  POOR | 9 |
| Andersson, C.M., Bjärås, G., Tillgren, P. and Ostenson, C-G.  (2005) (3) | Healthy lifestyle promotion; diabetes prevention. | Case study.  Longitudinal case studies of the three municipalities using different methods including the spidergram method, document analysis and group discussions. | Adults; children/young people; older people.  Three municipalities located in Stockholm County. | ***The Stockholm Diabetes Prevention Programme***: a comprehensive intervention based on principles of participation on different levels. The focus of this study is the assessment of the inter-sectoral participation in the programme, as a result little information given about the intervention itself. | Process | Qual 2  POOR | 6 |
| Boonekamp, G.M.M., Gutierrez-Sigler, M.D., Colomer, C. and Vaandrager, H.W.  (1996) (4) | Healthy lifestyle promotion. | Qualitative study.  30 stakeholder interviews were conducted. | Adults; children/young people; older people.  Valencia, Spain. | ***SUPER project***: to promote healthy eating behaviour. Key persons of organisations and institutions of eight sectors related to the food sector were interviewed: research, education, food production and distribution, the health sector, consumers, policy-makers, consumer organisations and mass media. | Process | Qual 3  POOR | 2 |
| Boonekamp, G.M.M., et al.  (1999) (5) | Healthy lifestyle promotion. | Qualitative study.  Interviews were conducted with representatives (n varies) of 13 cities included within the HC network. | Adults; children/young people.  Valencia, Spain.  13 cities within the 95 Valencian Community Healthy Cities Network (VCHCN). | ***WHO Europe Healthy Cities***: Healthy Cities aims to improve the health of the population. It does so through targeting health-related behaviours, raising health awareness and establishing health promotion strategies. | Process | Qual 3  POOR | 3 |
| Chomitz, V.R., et al.  (2010) (6) | Obesity; healthy lifestyle promotion. | Prospective cohort study.  Baseline (2004) to follow up (2007) evaluation design assessed change in children’s weight and fitness status. | Children/young people; BME, socioeconomic status.  Cambridge, MA, USA. | ***Healthy Living Cambridge Kids:*** The intervention included city policies and community awareness campaigns; physical education (PE) enhancements, food service reforms, farm-to-school-to-home programs; and family outreach and ‘BMI and fitness reports’. | Health +  Process | Quant 6  POOR | 4 |
| Cook, G., et al. (2009) (7) | Healthy lifestyle promotion.  Prevention of falls in the older community. | Case study.  One of five case studies. | Older people; educational level; social exclusion; disability.  Northumberland, UK. | ***Northumberland FISHNETS aims to keep older people FIT, INVOVED, SAFE & HEALTHY, through investment in sustainable community NETWORKS***. It is delivered through six linked task groups: community involvement; education and accreditation; home environment; physical activity and lifestyle; intermediate care; communications publicity and events.  A comprehensive falls prevention programme across Northumberland included:  public awareness campaigns; community rehabilitation team intervention; fall prevention exercise programmes; community exercise programmes and home improvement services. | Health +  Cost  Process | Qual 5  POOR TO MODERATE | 8 |
| Copeland, R., et al. (2011) (8) | Obesity; healthy lifestyle promotion. | Mixed methods evaluation, including audit of routinely collected data, cohort studies, nested studies, semi structured interviews with strand leads and board members as well as programme participants.  The collection of data was both cross-sectional and longitudinal. | Children/ young people.  Sheffield, UK. | ***Change 4 Life:*** the interventions were based on eight strands of work designed to achieve the strategic objectives:  1) A Breastfeeding Friendly City;  2) Parents as Positive Role Models for Healthy Eating;  3) Schools at the Heart of Healthy Communities;  4) Living Neighbourhoods;  5) Healthy Open Spaces;  6) Social Marketing - Change4Life;  7) Community Health Champions;  8) Cross Sector Innovation. | Wellbeing m  Health m  Cost  SDH + | Qual 5  POOR TO MODERATE  Quant 3  POOR | 10 |
| Cousins, J.M., et al.  (2011) (9) | Obesity.  Childhood obesity | Mixed methods evaluation.  Output data regarding the COPDP implementation was collected, as were changes in the community, partnerships facilitating the process and information upon potentially feasible long-term outcome measures. | Adults; children/young people.  North Carolina, USA.  North Carolina was ranked 11th in the nation for childhood obesity with almost 20% of all 10-17 year olds classified as obese. | ***Childhood Obesity Prevention Demonstration Project****:* Five communities within North Carolina delivered the COPDP in six settings: the community at-large, health care organisations, worksites, schools, child care centers, and religious organisations.. The interventions included: partnership development, built environment, farmer's markets, pediatric obesity clinical tools and training (for HCP), hospital worksite wellness programmes, in-school educational programmes and faith community intervention. | Process | Qual 0  POOR | 9 |
| de Groot, F.P., et al. (2010) (10) | Obesity.  Prevention and improvement in the prevalence of childhood obesity. | Mixed methods evaluation.  Three different data sources were used: Document analysis of intervention activities against a capacity building framework; Thematic analysis of key stakeholders; A quantitative Community Capacity Index Survey. | Children/young people.  Geelong, Victoria, Australia.  12,000 children aged 0-5. | ***Romp & Chomp****:* To increase the capacity of the Geelong community to promote healthy eating and active play and to achieve healthy weight in under 5s’. | Health +  Process | Qual 5  POOR TO MODERATE  Quant 3  POOR | 9 |
| de Leeuw, E., et al. (2015) (11) | Healthy lifestyle promotion.  Healthy Cities programme. | Mixed Methods Evaluation.  The evaluation of Phase V of the Healthy Cities takes a realist synthesis methodology and included case studies of the cities in the European Healthy Cities Network. The methodology is discussed elsewhere. | Cities in the WHO European Healthy Cities Network.  Europe. | ***WHO Europe Healthy Cities****:* The Healthy Cities programme with a focus in this case on governance within the European Healthy Cities Network. | Process | Quant 5  POOR | 8 |
| de Leeuw, E. et al. (2014) (12) | Healthy lifestyle promotion.  Healthy Cities programme. | Mixed methods evaluation.  This study takes a realist synthesis approach using a range of methods.  Data from 99 cities in the WHO European Healthy Cities Network and 31 national networks of healthy cities were collected on all elements of the programme logic by means of five instruments: the responses of cities throughout Phase V to the annual reporting template; a general evaluation questionnaire (online); three types of case studies; quantitative indicators mined from Eurostat and national data bases; and document analysis. | Cities within the WHO European Healthy Cities Network.  Europe. | ***WHO Europe Healthy Cities:*** Phase V has three core themes (caring and supportive environments, healthy living, and healthy urban environment and design) set within a durable framework of four overarching priorities 1) to address the determinants of health, equity in health and the principles of health for all; 2) to integrate and promote European and global public health priorities; 3) to put health on the social and political agenda of cities; and 4) to promote good governance and integrated planning for health). | Health +  Wellbeing ?  SDH +  Process | Qual 1  Quant 0  POOR | 10 |
| de Silva-Sanigorski, A.M. et al. (2010) (13) | Obesity  Reduce obesity and promote healthy eating and active play in children 0-5 years old. | Natural experiment.  The evaluation was repeat cross-sectional with a quasi-experimental design and comparison sample. | Children/young people.  Borough of Queenscliffe and City of Greater Geelong, Australia.  Targeted areas for intervention: Long Day Care Centres, Family Day Care Service, all pre-schools, Maternal Child Health Service, regional immunization services and community health services. | ***Romp & Chomp****:* The intervention consisted of a multi-setting, multi strategy approach with 8 project objectives and 4 key messages. Collaboration with existing health promotion activities; social marketing; training and resources for early childcare settings and workers; training and resources for health care workers; policy implementation and development; engagement with wider community. | Health +  Process | Quant 6  POOR | 9 |
| de Silva-Sanigorski, A., et al. (2011) (14) | Obesity.  Promotion of healthy eating and active play. | Natural experiment.  The evaluation had a cross-sectional, quasi-experimental design. An environmental audit was developed - a 45-item questionnaire capturing nutrition and physical activity-related aspects of the policy, socio-cultural and physical environments of the FDC service was completed by FDC care providers. Intervention (n = 28) and comparison (n = 223) samples. | Children/young people.  Geelong, Victoria, Australia.  Family Day Care Centres. | ***Romp & Chomp****:* Intervention to promote healthy eating and active play in early childhood settings. Development and distribution of promotional materials, newsletters and information cards; workshop and festival presence; support to early childcare settings; development and implementation of professional development, training and intervention strategies for early child care workers and health professionals; structured active play program, collaboration with other health promotion programs; integration of policies into handbooks, health service strategic and public health plans. | Health +  Process | Quant 4  POOR | 9 |
| Department of Health. (2010) (15) | Obesity. | Mixed methods evaluation.  Data was accrued throughout the year 2009 from multiple sources, including: surveys, interviews, participant feedback, click throughs (on webpage), programme costs, document review, case studies. | Adults; children/young people.  UK. | ***Change 4 Life****:* C4L promotes eight health-related behaviours through multi-level, multi-agency intervention work: 5-a-day, Sugar Swaps, Me Size Meals, Snack Check, Cut Back Fat, Meal Time, 60 Active Minutes, and Up and About. A social marketing approach has been at the core of C4L.  A six stage approach was taken to delivery: 1) Mobilising the Network; 2) Reframing the Issue; 3) Personalising the Issue; 4) Rooting the Behaviours; 5) Changing Social norms; 6) Supporting Change | Health +  Cost  Process | Quant 7  POOR | 8 |
| Donchin, M., et al. (2006) (16) | Healthy lifestyle promotion.  Healthy Cities Network. | Survey.  The survey covered six dimensions of Healthy Cities' principles and strategies, and each was analysed as a sum of scores of separate components and measures, converted to a 0-10 scale. | The Israel Network of Healthy Cities.  Israel.  The 18 cities in Israel that took part are active Healthy Cities. | ***Healthy Cities****:* Coordinators of 18 active Healthy Cities from the Israel Network of Healthy Cities. | Process | Quant 1  POOR | 3 |
| Eat Smart, Move More North Carolina Leadership Team. (2013) (17) | Obesity. | Survey.  3 surveillance systems were used:  • Behavioral Risk Factor Surveillance System (BRFSS)  • Child Health Assessment and Monitoring Program  (CHAMP)  • Youth Risk Behavior Survey (YRBS) | Adults; children/young people; older people.  North Carolina, USA. | ***Eat Smart, Move More****:* The North Carolina Obesity Prevention Plan 2007-2012 outlined strategies to make it easier for people to eat smart and move more. The strategies were organized into three areas: 1) Individuals and families; 2) Community and schools; 3) Policies and environments. | Health m | Quant 2  POOR | 7 |
| Economos, C.D., (2007) (18) | Obesity.  To improve the status of overweight and obesity amongst young children. | Non-randomised controlled trial.  One intervention city (Somerville) and two control cities within Massachusetts were included,. Measures were carried out on a total of 385 children in Somerville and 793 children in the control cities. | Children/young people.  Massachusetts, USA. | ***Shape Up Somerville***: aimed to increase the physical activity options of young people and the availability of healthy foods using a range of interventions which include: breakfast programmes, walking buses, school curriculum modifications, school policy developments, family events, parent outreach, city employee wellness programmes, farmers’ markets, local media placements and resource guides. The community, the school and the home were focused upon. The study lasted for three years within Somerville (2002 to 2005) | Health + | Quant 10  MODERATE TO GOOD | 8 |
| Farrington, J.L., et al. (2015) (19) | Obesity; smoking; alcohol; healthy lifestyle promotion.  Healthy Cities. | Mixed methods evaluation.  Combination of both a General Evaluation Questionnaire (GEQ) and Case Studies.  159 case studies were submitted (74 strategic, 79 thematic and 6 proudest achievement). Additionally, 71 of the HCP completed the GEQ. | Adults; children/young people.  Europe.  WHO European Healthy Cities Network.  By the end of 2013, a total of 99 member cities were included in the HCP network. Between the years of 2009-2013 was known as Phase V of the HCP. | ***WHO Europe Healthy Cities***: intervention varied between cities. Types of interventions discussed focused on preventing NCDs; tobacco, alcohol, healthy food and diet, active living for example. | Process | Qual 5  POOR TO MODERATE | 6 |
| Gantner, L.A. and Olson, C.M.  (2012) (20) | Obesity; healthy weight gain during pregnancy. | Mixed methods evaluation.  30 interviews and 31 pre/post surveys. | Females only.  New York, USA.  The HSP is implemented in a rural eight-county area of upstate New York. | ***Healthy Start Partnership****:* HSP worked collaboratively with multiple-agencies to offer pregnant women and post-partum women healthy weight -gain (during pregnancy) and -loss (post-partum) advice and support. Intervention activities included: a breastfeeding social marketing campaign, strengthened links between hospitals and community organisations which promote breastfeeding, work with local grocery stores to promote healthier food options near the checkout. | Process | Qual 5  POOR TO MODERATE | 3 |
| Goodwin, D.M., et al. (2013) (21) | Obesity.  Prevention and treatment of obesity. | Qualitative study.  Twenty interviews were conducted with local programme stakeholders and national policy implementers. | Adults; children/young people.  UK.  Nine Healthy Towns established within the UK during 2008. | ***Healthy Towns****:* 9 UK towns were allocated a share of £30m to implement a whole-town approach to improving the obesogenic environment. Towns were asked to be innovative in their approaches, and to ensure that they capture and collect data on the effectiveness of their approaches. | Process | Qual 7  GOOD | 2 |
| Goumans, M. and Springett, J.  (1997) (22) | Healthy lifestyle promotion.  Healthy Cities. | Qualitative study.  Semi-structured interviews with key informants from the ten cities (UK- and Holland- based). Questions related to five key topics of policy development and policy change. | Adults; children/young people.  Europe.  WHO European Healthy Cities. | ***WHO Europe Healthy Cities****:* The HC Project was initially established to implement the Health For All strategy at a local level. HC aim to target the following:  1) To bring together a network of European cities to assist and support the implementation of local HFA efforts. 2) To move health high on the political agenda. 3) To move health on the public agenda and integrate the health aspect into economy, culture and city life. 4) To develop city health policies. 5) To foster the development of supportive environments (physical and social). 6) To create action for health. 7) To facilitate the development of networks and communication skills. 8) To exchange knowledge and expertise. 9) Intersectoral collaborations; 10) Community participation; 11) Equity | Process | Qual 6  MODERATE TO GOOD | 10 |
| Green, G., et al. (2015) (23) | Healthy lifestyle promotion.  Healthy Cities. | Realist mixed methods evaluation.  Qualitative evidence from 112 highly structured case studies from 68 Network cities and 71 responses to a General Evaluation Questionnaire. | Adults; children/young people; BME and older people.  Cities within the WHO European Healthy Cities Network (EHCN).  Europe. | ***WHO Europe Healthy Cities****:* This study looks at Phase V of the WHO European Healthy Cities Network with specific focus on creating caring and supportive environments based on evidence from 68 network cities. Some reference to specific interventions can be found in the results section e.g. Dresden 'mobility and health in urban space' and Belfast 'child-friendly city' project. | Process | Qual 5  POOR TO MODERATE  Quant 2  POOR | 9 |
| Green, G., et al.  (2009) (24) | Healthy lifestyle promotion.  Healthy Cities. | Mixed methods evaluation.  Structured questionnaire from 44 cities, interviews with 24 city representatives, and examination of written publications from the project (1988 - 2003). | Cities within the WHO European Healthy Cities Network (EHCN).  Europe. | ***WHO Europe Healthy Cities:*** The Healthy Cities projects delivers a range of collaborative programs at both national and local levels of governance.  More specific interventions are not discussed here, rather a focus on the importance, development and maintenance of partnerships for health. | Process | Qual 1  Quant 2  POOR | 8 |
| Hoelscher, D.M. et al. (2010) (25) | Obesity.  Childhood obesity. | Cross-sectional study.  Examining regional changes in the prevalence of child obesity - using the School Physical Activity and Nutrition (SPAN) population-based surveillance data. | Children/young people.  Texas, USA.  Whole state and 8 Health Service Regions (HSR). | ***Several state-wide policies, initiatives, and legislative mandates to target child obesity***: (mandates for 135 min/week physical activity for elementary school children; establishing School Health Councils; implementing Texas Education Agency-approved coordinated school health programmes; and the Texas Public School Nutrition Program). E.g. a large, community-based health initiative, funded by the Paso del Norte Health Foundation that began with implementation of the Coordinated Approach To Child Health (CATCH) but also included community-level programs for nutrition and physical activity, as well as radio and television advertisements. | Health m | Quant 8  POOR TO MODERATE | 1 |
| Jeffery, R.W., et al. (1995) (26) | Obesity; reducing cardiovascular risk. | Non-randomised controlled trial.  One member of each of three pairs of matched communities received 7 years of community intervention activities. | Adults; children/young people.  Minnesota, USA. | ***Minnesota Heart Health Program****:* 7 years of community intervention activities including risk factor screening, mass media education, adult education classes, worksite interventions, home correspondence programmes, school-based programmes, restaurant programmes, point-of-purchase education in supermarkets. Weight gain prevention was emphasised for all adults and weight loss was encouraged among those who were obese or who had elevated risk factors known to be responsive to weight loss (e.g. high blood pressure). | Health - | Quant 6  POOR | 4 |
| Johnson, B.A., et al. (2012) (27) | Obesity.  Childhood obesity. | Non-randomised controlled trial.  Quasi-experimental, longitudinal design. | Adults; children/young people; socioeconomic status.  Victoria, Australia.  Intervention areas included 10 schools in Colac, Australia (4 pre-schools and 6 primary schools, n = 835). Comparator areas included 16 schools in Barwon SW Victoria, Australia (4 pre-schools and 12 primary schools, n = 977). | ***Be Active Eat Well****:* The intervention was delivered as a whole-of-community approach targeting 4 levels of socio-ecological model: 1) Community; 2) Workplace; 3) School; 4) Individual.  Interventions included: Nutritional - school-appointed dieticians, training for canteen staff in schools, community gardens, fast food outlet education; Physical Activity - after-school activities, walk to school days, sport club equipment; Screen time - education, newsletters, teacher training; and Widespread - media coverage, policy development, and incorporation of strategies into Health Promotion plans. | Health + | Quant 11  MODERATE TO GOOD | 7 |
| Kegler, M.C., et al. (2015) (28) | Obesity; smoking; healthy lifestyle promotion; chronic disease management. | Mixed methods evaluation.  Year 2 of a multisectoral, multiyear study. The evaluation included grantee interviews, organizational surveys, health council surveys, grantee progress reports. | Adults; children/young people; religious or cultural groups; educational level.  Missisipi Delta, USA. | ***Community based Policy, Systems and Environmental Change initiative to reduce risk factors for cardiovascular disease and stroke morbidity and mortality****:* The multisectoral multiyear community-driven initiative were delivered to support healthy behaviours through physical activity, nutrition, tobacco, chronic disease management and general health wellness interventions. | Wellbeing +  Health +  Process | Quant 0  POOR | 8 |
| Kegler, M.C., et al. (2008) (29) | Healthy lifestyle promotion. | Case study; qualitative study.  The CHCC was evaluated as 20 independent case studies that could then be compared between cases. Interviews with 28 co-ordinators and local staff which was supplemented by 9 focus groups. 16 interviews with community leaders were also conducted. | Adults; children/young people.  California, USA.  20 cities across California were included. The programmes delivered within each of the cities had to ensure that multi-sectoral work was completed and that the focus of said programmes was upon health improvement. | ***California Healthy Cities and Communities (Healthy Cities):*** The overall aim of the CHCC was to empower local communities/ organisations to improve the specified areas of health at a local level whilst also working to influence policy change. As part of the first year of funding, cities were required to solidify a multi-sectoral governance structure (i.e. get a team emplaced), and assess what the health needs of the city and community were. They finally had to produce a plan of actions for the following two years of funding. Years two and three were dedicated to programme implementation and evaluation. | Cost  Process | Qual 7  GOOD | 10 |
| Kegler, M.C., et al. (2009) (30) | Healthy lifestyle promotion. | Mixed methods evaluation; case study.  1) Coalition member surveys (n 243 to 330);  2) Key informant interviews (n=165);  3) Focus groups (n=26);  4) Programme document analysis (6 month and yearly progress reports). | Adults; children/young people.  California, USA.  See Kegler et al. (2008) | ***California Healthy Cities and Communities (Healthy Cities):****:* See Kegler et al. (2008). | Process | Qual 5  POOR TO MODERATE | 10 |
| Komro, K.A., et al. (2008) (31) | Alcohol.  To prevent the use of alcohol amongst adolescents. | Randomised controlled trial.  1) Intervention condition (multi-level intervention aimed at alcohol use prevention) and  2) Delayed onset condition (control).  The following outcomes were monitored: alcohol and drug use (via SR & parent/community leader survey) and alcohol purchasing attempts (via standardised protocol). | Adults; children/ young people.  Chicago, USA. | ***Project Northland Chicago****:* School-based Intervention: peer-led classroom curricula; Home-based Intervention: home-based educational sessions;  Community Intervention: peer leadership and youth planned projects, community organising and neighbourhood change. | Health m | Quant 12  GOOD | 6 |
| Larson, C.O., et al. (2009) (32) | Smoking. | Mixed methods evaluation.  Process data was collected through a web-based interactive system. Smoking prevalence data for North Nashville was monitored using data from a telephone survey conducted annually from 2001-2005 (across intervention years). | Adults; BME.  Nashville, Tennessee, USA.  REACH is implemented across the USA in a total of 42 communities. The results of this study refer to the community of Nashville. | ***Racial and Ethnic Approaches to Community Health (REACH) initiative***: aimed to educate, raise awareness and promote smoking cessation. Efforts were particularly targeted towards African Americans residing in Nashville, Tennessee - having a secondary aim of reducing health inequalities.  The programme worked at numerous levels, primarily across the community, individual and policy levels. Intervention was targeted at each level respectively. | Health +  Process | Quant 5  POOR | 8 |
| Liao, Y. L, et al.  (2010) (33) | Smoking. | Prospective cohort study.  42 local communities participated in the REACH project over 5 years. Data was collected cross-sectionally each year during the REACH study. No control groups used. | Adults; children/young people; BME; socioeconomic status.  USA. | ***Racial and Ethnic Approaches to Community Health (REACH) initiative***: REACH is a nation-wide project which empowers local communities to actively participate in the improvement of their own health. The project is grounded in the three principles previously mentioned, capacity building (coalitions which enable communities to work together), targeted actions (culturally tailored health promotion campaigns) and community and system change (influencing health-related policy and change within their local communities). | Health + | Quant 5  POOR | 8 |
| Lieberman, L., et al. (2013) (34) | Smoking. | Cross-sectional; survey. | Children/young people.  Rockland, New York City, USA. | ***Put It Out Rockland****:* The health department engaged consultants in a strategic planning process to build theory-based interventions. The PIOR interventions included school and community youth clubs based on New York State’s Reality Check; the Environmental Protection Agency’s smoke-free home promotion in preschools, daycare centers, and work sites; an award-winning cessation program with free nicotine replacement therapy; targeted local media campaigns; health care provider cessation resources; and partnering with municipalities and schools to implement and support smoke-free policies. These activities functioned within the larger context of strong statewide tobacco policies and cessation resources. Rockland also benefitted from media campaigns in the nearby New York City media market. | Health +  Cost  Process | Quant 2  POOR | 3 |
| Lipp, A., et al.  (2013) (35) | Healthy lifestyle promotion.  Healthy Cities. | Mixed methods evaluation.  Eight item questionnaire sent to all Healthy City partners. Questionnaire to be completed by a political representative of the Healthy City initiative. Questions related to the engagement and collaboration between sectors within each healthy city. | Adults; children/young people.  Worldwide: WHO Healthy Cities Network.  Questionnaire sent to all Healthy Cities worldwide. Respondents answered the questions with specific focus on the Healthy City that they are associated. 57 of the 79 cities included. | ***WHO Europe Healthy Cities****:* The HC network was established by the WHO in 1987. Since then, 79 cities have joined the network to be called a Healthy City (until 2008). HC offers member cities of the network advice on how to develop intersectoral collaboration as to improve the health of its population. | Process | Qual 4  POOR OT MODERATE  Quant 5  POOR | 5 |
| London Borough of Hackney.  (2014) (36) | Obesity; healthy lifestyle promotion. | Before and after study; survey.  Student BMI and waistline measurement at baseline and post-intervention; Physical activity performance levels at baseline, mid-point and post-intervention; A ‘Day in the Life’ behaviour and attitude questionnaires at baseline and post intervention; Parental behaviour and attitude questionnaires at baseline and post-intervention; Staff questionnaires at baseline and post-intervention. | Children/young people.  London Borough of Hackney, UK. | ***Change 4 Life – Health Heroes****:* A whole school approach to healthy weight. Interventions were planned across the four project strands: increasing physical activity (e.g. structured school programme); providing nutritional education and supporting access to healthy food (e.g. healthy lifestyle lessons, cook and eat sessions, Food Coop); and increasing the use of local parks and green spaces (e.g. PE in the park). An open approach was employed to trialling interventions as this was a pilot programme which enabled officers to assess impact and effectiveness of a range of interventions. | Wellbeing +  Health + | Quant 7  POOR | 10 |
| Lynn, W.R., et al. (1995) (37) | Smoking.  Smoking cessation. | Randomised controlled trial.  One community within each of 11 matched community pairs  (10 in the United States, 1 in Canada) was randomly assigned to intervention. | Adults.  USA and Canada.  COMMIT was centred on 11 participating research institutions and the corresponding local communities (11 matched community pairs -10 in the United States, 1 in Canada). | ***Community Intervention Trial for Smoking Cessation (COMMIT):*** Intervention focused on four primary channels: public education through the media and communitywide events; health care providers; work-sites and other organizations; and cessation resources. Within these channels, the protocol specified 58 mandated activities, defined so they could be carried out largely by community volunteers or local staff or agencies with limited external resources. | Health m | Quant 6  POOR | 9 |
| Maguire, M., et al. (2003) (38) | Alcohol and the reduction of alcohol related violence. | Mixed methods evaluation; Before and after study.  A TASC database was constructed for the project.  The police sources were incident records, custody handling records, crime records, CCTV logs, and extra information obtained by the analyst directly from officers.  These were supplemented by information from the hospital Accident and Emergency unit, | Adults; children/young people.  Cardiff, Wales, UK.  ‘Hot spots’ of  alcohol-related violence and disorder: | ***Tackling Alcohol Related Street Crime (TASC) project****:* Intervention categories were as follows:  1) Focused dialogue between the police and members of the licensed trade; 2) Measures aimed at improving the quality and behaviours of door staff; 3) Attempts made to influence licensing policy and practice; 4) Measures aimed at publicising the problem of alcohol-related violent crime; 5) Targeted policing operations directed at crime and disorder 'hot spots'; 6) A cognitive-behavioural programme for repeat offenders (named 'COV-AID'); 7) A training programme for bar staff (named 'Servewise'); 8) A programme of education about alcohol directed to school-age children; 9) Support for victims of alcohol-related assaults attending hospital. | Wellbeing +,m  Health m  Cost  Process | Quant 5  POOR | 6 |
| Martínez-Donate, A.P., et al. (2008) (39) | Smoking. | Cross sectional study.  The surveys were completed over the phone in San Diego (n = 1103), and a door-to-door survey was completed for Tijuana (n = 398) and Guadalajara (n = 400) due to low occurrence of home phones. | Adults; children/young people; BME.  San Diego; California, USA. Tijuana and Guadalajara, Mexico.  The CTCP is located within California, USA. | ***California Tobacco Control Program (CTCP):*** The CTCP introduced a $0.25 tax per pack at the beginning of the initiative. Additionally, the CTCP included: a mass media campaign on anti-tobacco; restrictions on tobacco advertising; smoking cessation programmes; introduced anti-smoking laws in California which were monitored by the CTCP; and enhanced school-based prevention programmes. Tijuana was moderately exposed to the CTCP as it was the border city between Mexico and California, thus many residents may have been affected by the CTCP. | Health + | Quant 9  POOR TO MODERATE | 2 |
| Mead, E.L., et al. (2013) (40) | Obesity; healthy lifestyle promotion. | Natural experiment; non-randomised controlled trial.  a quasi-experimental pre-/post-evaluation of Healthy Foods North conducted in three Inuit communities in one region of Nunavut and three Inuvialuit communities in one region of the Northwest territories. One remote community in each territory served as the comparison (“delayed intervention”) community and received the intervention post-evaluation. | BME.  Three Inuit communities in one region of Nunavut and three Inuvialuit communities in one region of the Northwest territories.  Nunavut and the Northwest territories, Canada (Canadian Arctic). | ***Healthy Foods North: a community-based multi-institutional nutritional and lifestyle intervention***: The 12-month program was developed from theory (social cognitive theory and social ecological models), formative research, and a community participatory process. It included an environmental component to increase healthy food availability in local stores and activities consisting of community-wide and point-of-purchase interactive educational taste tests and cooking demonstrations, media (e.g., radio ads, posters, shelf labels), and events held in multiple venues, including recreation centers and schools. | Health + | Quant 9  POOR TO MODERATE | 9 |
| Middleton, G. et al. (2014) (41) | Obesity. | Qualitative study.  29 interviews with stakeholders (Senior Health Officers [SWO] = 4, Community Members [CMs] = 13, and Public Health Workers [PHW] = 11). Semi-structured, one-to-one interviews completed with SHOs and CMs, and two focus groups carried out with PHWs. | Adults; children/young people; socioeconomic status.  Area in north-east of England, UK.  Multiple interventions delivered across all of the settings listed. As interventions were targeted at adults and children alike, both work places and schools were used to host interventions respectively. In total, three settings were targeted: community, school and the workplace. | ***Community based obesity prevention programme****:* 32 obesity prevention strategies and interventions were emplaced within the local area. These were to target both adults and children across multiple settings and through multiple approaches. For example, activity clubs were set up for children, nutrition education programmes for both adults and children, parkour, indoor bowling, and workplace challenges. The programme hoped to improve access to physical activity and educate/improve nutrition and food choices. | Process | Qual 6  MODERATE TO GOOD | 4 |
| Mikkelsen et al. (2016) (42) | Nutrition | Case study: comparison of three initiatives from around the world.  The 3 cases were presented as papers in a conference session on community-based interventions. The session identified key questions to be addressed across several domains. The lead investigator of each project created comparison tables, with successive rounds of review and comments by co-authors.  All 3 programmes were monitored by detailed formative or process evaluation. | Case 1: Promoting healthy eating and non-sedentary behaviour in the Local Community SoL-program (Denmark).  Case 2: Environmentally focused community randomised intervention trial for young child obesity prevention: Children’s Healthy Living (CHL) for Remote Underserved Minority Populations of the Pacific.  Case 3: B’More Healthy Communities for Kids (BHCK): a multi-level obesity prevention programme for low income urban African American children. | ***Local Community SoL program (Denmark); Children’s Healthy Living (Pacific); B’More Healthy Communities 4 Kids (USA):*** Mulit-level, multi-component approaches to community based interventions.  All three programmes were all integrated and coordinated programmes that intervene in multiple community settings/ institutions across the community environment in a synchronised manner and at the same time. Programmes differed slightly with respect to the nature of the interventions components due to differences in context and cultural traditions. | Process | Qual 5  POOR | n/a |
| Mohammadifard, N., et al. (2009) (43) | Healthy lifestyle promotion. | Non-randomised controlled trial.  See Sarrafzadegan et al. (2009) - This study was focused on the changes regarding nutritional quality. Therefore, it only used the cross sectional questionnaire data from 5 years of intervention/ control exposure. | Adults; children/young people.  Iran.  See Sarrafzadegan et al. (2009) | ***Isfahan Healthy Heart Program****:* See Sarrafzadegan et al. (2009). | Health + | Quant 7  POOR | 6 |
| Paschall, M.J. et al. (2009) (44) | Smoking; drug use. | Non-randomised controlled study.  Quasi-experimental design. Data was used from a state-wide, bi-annual Youth Risk Behaviour Survey administered in over 90% of the state’s schools. 23 communities in Vermont formed a coalition as part of the ND programme, the impact of which could be assessed against areas without the ND programme. Only data from the survey was used here. | Adults; children/young people.  Vermont, USA.  23 ND coalitions aimed to deliver intervention across multiple settings with the ambition of increasing awareness of substance abuse, and improving the levels of substance abuse in youths. As such, interventions were targeted at schools, the community, and the family. | ***New Directions****:* ND coalitions implemented a variety of programs, to reduce alcohol and other substance abuse, that include school-based prevention curricula, student assistance programs, mentoring, substance-free alternative activities, and family outreach programs. Coalition activities also included public awareness campaigns and other environmental strategies, and they served to enhance collaboration and networking among community organisations. | Health + | Quant 4  POOR | 1 |
| Peters, J. et al.  (2005) (45) | Obesity.  Focus on healthy eating. | Case study.  Six New Deal for Communities (NDC) Healthy Eating Initiative case studies. Multiple visits to each NDC, additional communication, written material from NDC and a survey of the existing evidence-base for healthy eating initiatives from the published literature. | Adults; children/young people; older people; socioeconomic status.  UK.  The six case studies are set in New Deal for Communities in  Bristol, Middlesbrough, Oldham, Salford, Southampton and Walsall. | ***New Deal for Communities****:* The six NDCs in the case studies identified their local needs. Individual approaches from the case studies include:  - effective prevention of obesity and increased weight through healthy eating interventions;  *-* activities: Two NDC are addressing healthy eating with one activity each (allotments, weight management)  Four NDC are addressing healthy eating through larger projects (e.g. Healthy Hearts project, health and wellbeing project that includes poor diet and nutrition within it) | Health m | Qual 1  POOR | 7 |
| Pettigrew, S. et al. (2014) (46) | Obesity.  Preventing and reducing childhood obesity | Qualitative study.  An online survey was distributed to the 25 EPODE programmes running worldwide. Eighteen programmes responded from a total of 14 countries. | Adults; children/young people.  Worldwide.  The EPODE programme is delivered in 25 locations across the world. EPODE intends to engage a broad range of stakeholders who will collaboratively work to prevent childhood obesity. | ***Together Let’s Prevent Childhood Obesity”***  ***(Ensemble Prévenons l’Obésité des Enfants (EPODE):*** EPODE advocates that its' partners implement a co-ordinated, capacity building approach which works with multiple stakeholders and within multiple settings. The approach is adaptable to local contexts. Each EPODE area has a designated project co-ordinator to facilitate the flow of information between partners. | Process | Qual 7  GOOD | 8 |
| Pierce, J.P., et al. (1998) (47) | Smoking. | Before and after study. | Adults; children/young people.  California, USA.  The CTCP is located within California, USA. The approach however works at multiple levels, including policy implementation, intervention delivery and school-based prevention programmes. | ***California Tobacco Control Program****:*  The initiative mandated funding for mass media anti-tobacco campaigns, local health agencies to provide technical support and monitor adherence to antismoking laws, community-based interventions selected by a competitive grants process, and enhancement of school based prevention programs. | Health + | Qual 6  MODERATE TO GOOD | 2 |
| Plumer K D, et al. (2010) (48) | Healthy lifestyle promotion. | Mixed methods evaluation.  47 co-ordinators in 52 areas completed the survey. Questions on the survey related to the following:  - Local project characteristics  - Staffing, co-ordination and resource  - Resource available to Healthy Cities Offices  - Conceptual quality  - Network integration  - Level of evaluation activity  - Achieved success. | Adults, children/young people.  Germany.  The HCN was, as of 2002, operating in 52 municipalities across Germany - broadly divided into East and West Germany. Each of the municipalities strived to work towards nine common actions (See intervention description section). The HCN however works at multiple levels and ensures that multiple-agencies are collaboratively involved in the project. | ***WHO Europe Healthy Cities***: Further information not provided in the paper, however the municipalities need to align to the nine-point programme of actions and additionally the three core objectives. | Process | Quant 5  POOR | 10 |
| Public Health England.  (2014) (49) | Obesity; healthy lifestyle promotion. | Case study.  Fourteen case studies were presented within the document. Of the fourteen, two are considered here:  - Food and Cornwall (Cornwall), and  - Breaking the Intergenerational Cycle of Obesity (Gloucestershire). | Adults; children/young people.  Cornwall and Gloucestershire, UK.  Food and Cornwall (Cornwall) was delivered across economic, environmental, social and political systems to increase access to healthful food across the population.  Breaking the intergenerational cycle of obesity (Gloucestershire) was targeted at three levels: 1) community level; 2) operational level; and, 3) strategic level. | ***System Leadership Programmes****:*  Food and Cornwall: the work is targeted at vulnerable groups and communities reached through place based approaches that bring together families, community groups, charities and people using and providing emergency food provision and support. Activities include: short films, school-based food policy, distribution of surplus food, education workshops, lunch clubs, and support for catering standards in various settings.  Gloucestershire, breaking the intergenerational cycle of obesity: The programme in Gloucestershire looks to enable behaviour change at the community level, to identify and work with relevant systems to develop a shared plan for healthier lifestyle behaviours at an operational level, and to develop system leadership across the partner agencies at a strategic level. | Process | Qual 2  POOR | 10 |
| Raine, K.D. et al. (2013) (50) | Obesity; alcohol; healthy lifestyle | Before and after study.  Separate samples of adults in Healthy Alberta Communities (HAC) were surveyed before and after the interventions and compared responses to identical survey questions asked of adults living in Alberta in two waves of the Canadian Community Health Survey (CCHS). Physical measurements; and blood biochemistry were also measured in samples from HAC communities, but a comparison group was not available for these. | Adults; older people; BME; social exclusion; socioeconomic status.  Alberta, USA.  4 Healthy Alberta Communities (HAC) in Canada were selected for the intervention: Bonnyville (town), St. Paul (rural), Norwood/ North Central Edmonton (inner city) and Medicine Hat (small city).  Each community presented an opportunity for developing unique intervention approaches to chronic disease prevention. | ***Healthy Alberta Communities****:* Each of the 4 HACs presented an opportunity for developing unique intervention approaches to chronic disease prevention.  Each community to develop community-specific interventions relevant to their assessed needs. Community priorities for action were identified to supplement ongoing opportunistic interventions. In all communities, a number of inter-related initiatives were implemented with participation of community stakeholders, and coordination by HAC community coordinators. | Wellbeing +  Health –  SDH + | Quant 3  POOR | 6 |
| Ritsatakis, A., et al. (2015) (51) | Healthy lifestyle promotion; health inequalities.  Healthy Cities. | Mixed methods evaluation.  Data sources used within the evaluation framework:  General Evaluation Questionnaire (specifically to evaluate phase V);  Annual Reporting Template;  Case studies (qualitative analysis used);  City Health Profiles;  Equity sub-network. | Adults, children/young people.  Europe.  Cities within WHO European Healthy Cities Network | ***WHO Europe Healthy Cities****:* Cities within the European Healthy Cities Network signed up to tackle the social determinants of inequalities in health through a range of city specific interventions.  Examples from the case studies included:  - In Vienna (Austria) a project involving caterers, teachers and parents to improve nutrition in kindergartens in areas with poor child nutrition.  - In Dresden (Germany), Ostfold (Norway) and Preston (England) projects to encourage physical activity focused on individual behaviour change with urban design and involving the education system.  The results section of the paper include examples of cities tackling social determinants of inequalities in health including:  - In Liverpool (England) improvement in poor housing to reduce respiratory disease;  - In Montijo (Portugal) food assistance for disadvantaged groups;  - in Preston (England) community street audits and asset mapping;  - In Ostfold (Norway) influencing national policy to tackling inequalities through the Norwegian national healthy cities network. | Process | Qual 2  Quant 3  POOR | 10 |
| Samuels, S.E. et al. (2010) (52) | Obesity; obesity related type II diabetes; healthy lifestyle promotion. | Mixed methods evaluation; case study.  Six sites each making up one case study. | Children/young people; socioeconomic status.  California, USA.  The HEAC programme works across multiple settings and sectors (policy, state, community, city and institutional). Six Californian communities were selected to be HEAC towns (Baldwin Park, Chula Vista, Oakland, Santa Ana, South Shasta County, and South Los Angeles). | ***California Endowment’s Health Eating Active Communities (HEAC) program****:* HEAC is a $26m investment across 4 years. HEAC has a two pronged approach. The first prong (community) is to create policy and environmental changes which will increase access to healthy foods and PA - this targets and supports community members and community sectors. The second prong (technical assistance, advocacy and policy) seeks to build and support state-wide policy reforms which will improve food and PA environments. | Process | Quant 5  POOR | 6 |
| Sanigorski, A.M. et al..(2008) (53) | Obesity.  Reducing unhealthy weight gain in children. | Non-randomised controlled trial.  Quasi-experimental, longitudinal design with anthropometric and demographic data collected on Colac children in four preschools and six primary schools at baseline (2003, n=1001) and follow-up (2006, n=839). A comparator sample was also collected from a stratified, random sample of 16 pre- and primary- schools (2003, n=1183; 2006, n=979). | Adults; children/young people; socioeconomic status.  Victoria, Australia.  See Johnson et al. (2012). | ***Be Active Eat Well****:*  See Johnson et al. (2012). | Health + | Quant 12  GOOD | 7 |
| Sarrafzadegan, N. et al. (2009) (54) | Healthy lifestyle promotion. | Non-randomised controlled trial.  Community-wide trial conducted in two intervention counties (Isfahan and Najaf-Abad) and a control area (Arak). Baseline, midpoint and post-intervention evaluation design. | Adults; children/young people.  Iran.  Three different communities: Isfahan and Najaf-Abad were intervention communities, Arak a control community (evaluated only, no intervention). | ***Isfahan Healthy Heart Program****:* IHHP is a number of comprehensive community-based lifestyle interventions targeting diet, physical activity, smoking behaviour and stress management. Interventions are targeted to individuals, populations and the environment (dependent on needs assessment). IHHP delivers 10 interventions across multiple-sectors - each intervention targets either individuals, populations or the environment. | Health m | Quant 7  POOR | 9 |
| Sautkina, E. et al. (2014) (55) | Obesity.  Obesity prevention. | Qualitative study.  Semi-structured interviews with 20 informants, purposively selected to represent national and local programme development, management and delivery were undertaken. | Adults; children/young people; older people.  England, UK.  Nine bid-winning English ‘healthy towns’. These comprised:  A London borough (Tower Hamlets), three large cities (Manchester, Portsmouth and Sheffield), two medium-sized town (Halifax and Middlesbrough), one metropolitan borough (Dudley) and two smaller provincial towns (Tewkesbury and Thetford). | ***Healthy Towns****:* Overall, the nine towns implemented in excess of 300 individual interventions, primarily focused on promoting a healthy diet and increasing physical activity. | Process | Qual 7  GOOD | 1 |
| Schoen, M.W. et al. (2014) (56) | Obesity; smoking. | Network analysis study | Adults; children/young people.  Missouri, USA.  Each of the three programmes were operating within multiple communities across Missouri. One community had two programmes running simultaneously, the remainder of the communities only had one programme running within it. | The three public health funding programmes included:  1) ***Social Innovation for Missouri (SIM):*** aimed to address the public health goals of tobacco cessation and obesity prevention through the development of partnerships with key stakeholders in local municipalities, rural, and urban neighborhoods. Also sought to increase community capacity building i.e. implemented a WSA to tackling these two health issues.  2) ***Tobacco Prevention & Cessation Initiative (TPCI):*** focus on tobacco control only.  3) ***Healthy & Active Communities Initiative (H&AC):*** focus on obesity prevention only. | Process | Quant 2  POOR | 4 |
| Schuit, A.J. et al.  (2006) (57) | Cardiovascular disease (CVD) risk factor reduction.  Physical activity, dietary fat intake, and tobacco smoking. | Prospective cohort study.  The study used two cohort populations, one from the Hartslag Limburg population and one from a reference population (no intervention delivered). As such, the data of 3000 subjects (selected via a gender- and age- stratification) and 2414 subjects were collected at baseline and 5 years respectively for the project cohort. For the reference region, data of 895 subjects and 758 subjects were collected accordingly at the two time points. | Adults; children/young people; socioeconomic status.  Holland.  Interventions within the project were delivered across numerous settings in the province of Limburg, Maastricht. | ***The Hartslag Limburg project*** had two broad strategies. One, to deliver a population-wide strategy to target CVD risk factors - this was highly focused on low SES communities. Two, to deliver a subgroup strategy to assist those with CVD or CVD risk factors. To do so, a large volume of interventions (n = 790, 590 of which classed as major interventions) were carried out across multiple levels and through multiple agencies. Interventions included: computer-tailored nutrition education, nutrition education tours in supermarkets, public–private collaboration with the retail sector, television programs, food labelling, smoke free areas, creating walking and bicycling clubs, walking and cycling campaigns, and a stop-smoking campaign. | Health -, m | Quant 8  POOR TO MODERATE | 7 |
| Schulz, A.J. et al. (2005) (58) | Obesity; healthy living lifestyle promotion; diabetes prevention. | Case Study. | Adults; BME; females only; social exclusion, socioeconomic status; disability.  Detroit, Michigan, USA.  Community based project. | ***Healthy Eating and Exercising to Reduce Diabetes project****:* Engaging the communities in a voluntary participation project increasing education and awareness of diabetes. The focus was on healthy eating, reduction of obesity and physical exercise, reducing disability and decreasing the mortality rate as a direct result of diabetes.  Interventions were also designed to help with the formation of connections between social and health factors and diabetes. | Wellbeing m  Health +  Cost  SDH m  Process | Quant 2  POOR | 1 |
| Schwarte, L. et al. (2010) (59) | Obesity.  Obesity prevention. | Mixed methods evaluation.  Methods used: logic models; public health department environmental assessments; farmers market and produce stand environmental assessments; physical activity/built environment assessment; community resident focus groups; elected governmental official stakeholder interviews; grantee reporting interviews and profiles; community resident survey and policy maker survey. | Adults, children/young people; social exclusion; socioeconomic status.  Central California, USA.  Rural and deprived regions of California (Fresno, Kings Kern, Madera, Tulare, Merced, Stanislaus and San Joaquin). | ***Central California Regional Obesity Prevention Program (CCROPP):*** Varied between regions but included: improving public health department capacity; community engagement and partnership working, changes to nutrition and physical activity environments and policy change. | Health +  Process | Qual 1  POOR | 10 |
| Serpas, S. et al. (2013) (60) | Obesity. | Case study.  San Diego Healthy Weight Collaborative. | Children/young people; BME; socioeconomic status.  San Diego, USA. | ***San Diego Healthy Weight Collaborative (SDHWC):*** one of 10 teams selected by the National Initiative for Children’s Healthcare Quality (NICHQ) and Health Resources and Services Administration (HRSA) to develop systems approaches to address childhood obesity. | Process | Qual 1  POOR | 10 |
| Simos, J. et al.  (2015) (61) | Healthy Lifestyle Promotion.  Healthy Cities. | Mixed methods evaluation.  Realist evaluation framework using a triangulation of methods:  - General Evaluation Questionnaire (GEQ)  - Case study templates.  - HIA factor analysis. | Adults; children/young people.  Europe.  The European Healthy Cities Network. | ***WHO Europe Healthy Cities***: Health Impact Assessment in phase V of the Healthy Cities project. | Process | Quant 0  POOR | 10 |
| Stafstrom, M. and Larsson, S.  (2007) (62) | Alcohol. | Qualitative study.  Unstructured focused interviews (n = 12), focus group discussions (n = 5), and formative discussion seminars (n = 2) were held to capture data addressing the study aim. Data was also derived from steering group minute notes, and session observations. | Adults; children/young people.  Trelleborg, Sweden. | ***Trelleborg project****:* aimed to reduce alcohol consumption and related harm. A number of interventions were delivered cross-sectorally with the intent of achieving the four objectives. The interventions included: school action plans on alcohol and drug use (which included curricula based activities; educational programmes for parents; inception of parental networks; influence drug and alcohol prevention policies in workplaces; intervention aimed at convenience stores to reduce imported tobacco and alcohol distribution. | Process | Qual 3  POOR | 3 |
| Stead, M. et al.  (2001) (63) | Drug use. | Non-randomised controlled trial.  Ten schools, six experimental and four controls, participated in a longitudinal quasi-experimental study. Half of the experimental schools received a `full’ version of the Year Nine intervention (i.e. all the components, for both primary and secondary targets), and half received a `partial’ version of the intervention (components for the primary target of Year Nine pupils only). This element of the experimental design was intended to assess the value of secondary targeting. The four control schools did not receive the intervention, but received normal levels of school and community drugs education and prevention activity, which were monitored throughout. | Children/young people.  Northumbria, UK.  School, media & local community, implemented by Northumbria Drugs Prevention Team | ***NE Choices****:* aimed to prevent adolescent drug use*.* In the Year Nine intervention, the interpersonal components, which formed the core of the intervention, were:  - Drama workshop, delivered by actors from a professional theatre company to classes of 25 pupils, involving a play and `Forum Theatre’ role-play in which different behavioural responses to drug offer situations were `rehearsed’. The content of the drama workshop was partly based on exploratory research into typical drug offer situations in the region.  - `Friday Forum’, an interactive information session for a smaller group of Year Nine pupils within each school. The intention was to reinforce the drama workshop by equipping this smaller group of pupils with drugs prevention information which could be disseminated to their peers either informally or through classroom work. The Forum was also facilitated by the actors.  - Classroom follow-up to the drama in Personal, Social and Health Education (PSHE) lessons, delivered by teachers using a Teachers’ Pack based on the play.  - Parents’ session, comprising elements of the drama workshop, information and discussion. The session was open to all parents of Year Nine pupils in each school*.* | Health m  Process | Quant 7  POOR | 3 |
| Wagenaar, A.C. et al. (1999) (64) | Alcohol. | Mixed methods evaluation; randomised controlled trial; qualitative study.  Random sample of seven communities.  Self-administered surveys of children in 9th (14-15 years old) grade, 12th grade (17-18 years old) (baseline and follow-up for 12th graders).  Additionally, telephone surveys with young adults between 18-20 (3095 baseline and 1721 at follow-up); telephone surveys with managers of alcohol retail outlets (502 baseline and 556 follow-up), and conducted pseudo-underage alcohol buying attempts.  Conducted content analysis of newspaper coverage related to alcohol issues, a collection of archival data of community-level indicators such as arrests and car crashed and a variety process evaluation data. | Adults; children/young people.  USA. | ***Communities Mobilizing for Change on Alcohol (CMCA)****:* Intervention was in the form of policy change and working with the communities involved to change attitudes toward underage drinking. | Wellbeing ?  Health +  SDH ?  Processs | Quant 10  MODERATE TO GOOD | 10 |
| Werna, E. and Harpham, T.  (1996) (65) | Healthy lifestyle promotion.  Healthy Cities. | Qualitative study.  In-depth interviews with 47 key actors in the Chittagong HCP. The interviews were bolstered by observations of the actors, and additionally, informal conversations with citizens. | Adults; children/young people.  Chittagong, Bangladesh.  A HCP located in Chittagong, Bangladesh. Chittagong is the second largest city in Bangladesh, with a population (1993) between 1.5 and 2.5 million. Bangladesh was considered to be a developing country and the HCP model had not yet been implemented in such a country. The original HCP model was intended to be implemented in urban and industrialised areas.  Many of the areas within Chittagong are in poverty, with approximately 1 million living in such conditions. Health services are scarce in the city. | ***Healthy Cities****:* Based on the WHO HCP model, the HCP Chittagong project was established to ameliorate the health of the city. This included work with multiple agencies (as aforementioned), meetings and workshops between partners, and intervention targeted at specific population groups, levels, and policies. | Process | Qual 3  POOR | 8 |

1. Active Living by D. Growing a movement: healthy kids, healthy communities. Final report. Chapel Hill, Ca: Active Living by Design, 2014 2014. Report No.

2. Amed S, Shea S, Pinkney S, Wharf Higgins J, Naylor P-J. Wayfinding the Live 5-2-1-0 Initiative-At the Intersection between Systems Thinking and Community-Based Childhood Obesity Prevention. International Journal Of Environmental Research And Public Health. 2016;13(6).

3. Andersson CM, Bjärås G, Tillgren P, Ostenson CG. A longitudinal assessment of inter-sectoral participation in a community-based diabetes prevention programme. Social Science & Medicine. 2005;61:2407-22.

4. Boonekamp GMM, GutierrezSigler MD, Colomer C, Vaandrager HW. Opportunities for health promotion: The knowledge and information system of the Valencian food sector, Spain. Health Promotion International. 1996;11:309-19.

5. Boonekamp GMM, Colomer C, Tomas A, Nunez A. Healthy Cities Evaluation: the co-ordinators perspective. Health Promotion International. 1999;14:103-10.

6. Chomitz VR, McGowan RJ, Wendel JM, Williams SA, Cabral HJ, King SE, et al. Healthy living Cambridge kids: a community-based participatory effort to promote healthy weight and fitness. 2010;18(suppl 1):s45-s53.

7. Cook G, Dawson P, Elliot D. Case study 1: explicating the role of partnerships in Northumberland FISHNETS. In: Holland K, Warne AR, Howath ML, editors. Explicating the role of partnerships in changing the health and well-being of local communities in urban regeneration areas: an evaluation of the Warnwarth Conceptual framework: a case study approach. 32009. p. 14-34.

8. Copeland R, Moullin M, Reece L, Gibson D, Barrett D. Sheffield's-let's Change4Life: a whole systems approach to tackling overweight and obesity in childre, young people and families. A local evaluation report. Sheffield: Sheffield Hallam University, 2011 2011. Report No.

9. Cousins JM, Langer SM, Rhew LK, Thomas C. The Role of State Health Departments in Supporting Community-Based Obesity Prevention. Preventing Chronic Disease. 2011;8(10):A87.

10. de Groot FP, Robertson NM, Swinburn BA, de Silva-Sanigorski AM. Increasing community capacity to prevent childhood obesity: challenges, lessons learned and results from the Romp & Chomp intervention. BMC Public Health. 2010;10:8.

11. de L, Kickbusch I, Palmer N, Spanswick L. European Healthy Cities come to terms with health network governance. Health Promotion International. 2015;30:i32-i44.

12. de Leeuw E, Tsouros AD, Dyakova M, Green G, eds. Healthy cities: promoting health and equity: evidence for local policy and practice. Copenhagen: WHO Regional Office for Europe; 2014 2014.

13. de Silva-Sanigorski AM, Bell AC, Kremer P, Nichols M, Crellin M, Smith M, et al. Reducing obesity in early childhood: results from Romp & Chomp, an Australian community-wide intervention program. American Journal of Clinical Nutrition. 2010;91:831-40.

14. de Silva-Sanigorski A, Elea D, Bell C, Kremer P, Carpenter L, Nichols M, et al. Obesity prevention in the family day care setting: impact of the Romp & Chomp intervention on opportunities for children's physical activity and healthy eating. Child: Care, Health and Development. 2011;37:385-93.

15. Department Of H. Change4life: one year on2010 2010.

16. Donchin M, Shemesh AA, Horowitz P, Daoud N. Implementation of the Healthy Cities' principles and strategies: An evaluation of the Israel Healthy Cities Network. Health Promotion International. 2006;21:266-73.

17. Eat Smart Move More North Carolina Leadership T. Eat Smart, Move More: North Carolina’s Plan to Prevent Overweight, Obesity and related chronic diseases, 2007-2012: Final Report. Raleigh, NC: Eat Smart Move More; 2013 2013.

18. Economos CD, Hyatt RR, Goldberg JP, Must A, Naumova EN, Collins JJ, et al. A community intervention reduces BMI z-score in children: Shape Up Somerville First Year Results. Obesity. 2007;15(5):1325-36.

19. Farrington JL, Faskunger J, Mackiewicz K. Evaluation of risk factor reduction in a European City Network. Health Promotion International. 2015;30:i86-i98.

20. Gantner LA, Olson CM. Evaluation of public health professionals' capacity to implement environmental changes supportive of healthy weight. Evaluation and Program Planning. 2012;35:407-16.

21. Goodwin DM, Cummins S, Sautkina E, Ogilvie D, Petticrew M, Jones A, et al. The role and status of evidence and innovation in the healthy towns programme in England: a qualitative stakeholder interview study. Journal of Epidemiology and Community Health. 2013;67:106-12.

22. Goumans M, Springett J. From projects to policy: 'Healthy Cities' as a mechanism for policy change for health? Health Promotion International. 1997;12:311-22.

23. Green G, Jackisch J, Zamaro G. Healthy cities as catalysts for caring and supportive environments. Health Promotion International. 2015;30:i99-i107.

24. Green G, Price C, Lipp A, Priestley R. Partnership structures in the WHO European Healthy Cities project. Health Promotion International. 2009;24:37-44.

25. Hoelscher DM, Kelder SH, Pérez A, Day RS, Benoit JS, Frankowski RF, et al. Changes in the regional prevalence of child obesity in 4th, 8th, and 11th grade students in Texas from 2000–2002 to 2004–2005. Obesity. 2010;18(7):1360-8.

26. Jeffery RW, Gray CW, French SA, Hellerstedt WL, Murray D, Luepker RV, et al. Evaluation of weight reduction in a community intervention for cardiovascular-disease risk - changes in body-mass-index in the Minnesota Heart health program. International Journal of Obesity. 1995;19:30-9.

27. Johnson BA, Kremer PJ, Swinburn BA, de Silva-Sanigorski AM. Multilevel analysis of the Be Active Eat Well intervention: environmental and behavioural influences on reductions in child obesity risk. International Journal of Obesity. 2012;36:901-7.

28. Kegler MC, Honeycutt S, Davis M, Dauria E, Berg C, Dove C, et al. Policy, Systems, and Environmental Change in the Mississippi Delta: Considerations for Evaluation Design. Health Education & Behavior. 2015;42:57S-66S.

29. Kegler MC, Norton BL, Aronson R. Achieving organizational change: findings from case studies of 20 California healthy cities and communities coalitions. Health Promotion International. 2008;23:109-18.

30. Kegler MC, Painter JE, Twiss JM, Aronson R, Norton BL. Evaluation findings on community participation in the California Healthy Cities and Communities program. Health Promotion International. 2009;24:300-10.

31. Komro KA, Perry CL, Veblen-Mortenson S, Farbakhsh K, Toomey TL, Stigler MH, et al. Outcomes from a randomized controlled trial of a multi-component alcohol use preventive intervention for urban youth: Project Northland Chicago. Addiction. 2008;103:606-18.

32. Larson CO, Schlundt DG, Patel K, Wang H, Beard K, Hargreaves MK. Trends in Smoking Among African-Americans: A Description of Nashville's REACH 2010 Initiative. Journal of Community Health. 2009;34:311-20.

33. Liao YL, Tsoh JY, Chen R, Foo MA, Garvin CC, Grigg-Saito D, et al. Decreases in Smoking Prevalence in Asian Communities Served by the Racial and Ethnic Approaches to Community Health (REACH) Project. American Journal of Public Health. 2010;100:853-60.

34. Lieberman L, Diffley U, King S, Chanler S, Ferrara M, Alleyne O, et al. Local Tobacco Control: Application of the Essential Public Health Services Model in a County Health Department's Efforts to Put It Out Rockland. American Journal of Public Health. 2013;103:1942-8.

35. Lipp A, Winters T, de L. Evaluation of Partnership Working in Cities in Phase IV of the WHO Healthy Cities Network. Journal of Urban Health-Bulletin of the New York Academy of Medicine. 2013;90:S37-S51.

36. London Borough of H. Health Heroes. A whole school approach to healthy weight. 2014.

37. Lynn WR, Freedman LS, Green SB, Corle DK, Gail M, Glasgow RE, et al. COMMUNITY INTERVENTION TRIAL FOR SMOKING CESSATION (COMMIT) .1. COHORT RESULTS FROM A 4-YEAR COMMUNITY INTERVENTION. American Journal of Public Health. 1995;85:183-92.

38. Maguire M, Nettleton H, Rix A, Raybould S. Reducing alcohol-related violence and disorder: an evaluation of the 'TASC' project (Home Office research study 265). London: Home Office, 2003 2003. Report No.

39. Martínez-Donate AP, Hovell MF, Hofstetter CR, Gonzélez-Pérez GJ, Kotay A, Adams MA. Crossing Borders: The Impact of the California Tobacco Control Program on Both Sides of the US--Mexico Border. American Journal of Public Health. 2008;98:258-67.

40. Mead EL, Gittelsohn J, Roache C, Corriveau A, Sharma S. A Community-Based, Environmental Chronic Disease Prevention Intervention to Improve Healthy Eating Psychosocial Factors and Behaviors in Indigenous Populations in the Canadian Arctic. Health Education & Behavior. 2013;40:592-602.

41. Middleton G, Henderson H, Evans D. Implementing a community-based obesity prevention programme: experiences of stakeholders in the north east of England. Health Promotion International. 2014;29:201-11.

42. Mikkelsen BE, Novotny R, Gittelsohn J. Multi-Level, Multi-Component Approaches to Community Based Interventions for Healthy Living-A Three Case Comparison. International Journal Of Environmental Research And Public Health. 2016;13(10).

43. Mohammadifard N, Kelishadi R, Safavi M, Sarrafzadegan N, Sajadi F, Sadri GH, et al. Effect of a community-based intervention on nutritional behaviour in a developing country setting: the Isfahan Healthy Heart Programme. Public Health Nutrition. 2009;12:1422-30.

44. Paschall MJ, Flewellng RL, Grube JW. Using statewide youth surveys to evaluate local drug use policies and interventions. Contemporary Drug Problems. 2009;36:427-45.

45. Peters J, Ellis E, Goyder G, Blank L. Healthy eating initiatives: case studies (New Deal for Communities National Evaluation research report 56). Sheffield: Sheffield Hallam University; 2005 2005.

46. Pettigrew S, Borys JM, du P, H R, Walter L, Huang TTK, et al. Process evaluation outcomes from a global child obesity prevention intervention. BMC Public Health. 2014;14:10.

47. Pierce JP, Gilpin EA, Emery SL, White MM, Rosbrook B, Berry CC. Has the California Tobacco Control Program reduced smoking? Jama-Journal of the American Medical Association. 1998;280:893-9.

48. Plumer KD, Kennedy L, Trojan A. Evaluating the implementation of the WHO Healthy Cities Programme across Germany (1999-2002). Health Promotion International. 2010;25:342-54.

49. Public Health E. Paths to public health and wellbeing: examples of local authority action in the South-West. London: Public Health England, 2014 2014. Report No.

50. Raine KD, Plotnikoff R, Schopflocher D, Lytvyak E, Nykiforuk CIJ, Storey K, et al. Healthy Alberta Communities: Impact of a three-year community-based obesity and chronic disease prevention intervention. Preventive Medicine. 2013;57:955-62.

51. Ritsatakis A, Ostergren PO, Webster P. Tackling the social determinants of inequalities in health during Phase V of the Healthy Cities Project in Europe. Health Promotion International. 2015;30:i45-i53.

52. Samuels SE, Craypo L, Boyle M, Crawford PB, Yancey A, Flores G. The California Endowment's Healthy Eating, Active Communities Program: A Midpoint Review. American Journal of Public Health. 2010;100:2114-23.

53. Sanigorski AM, Bell AC, Kremer PJ, Cuttler R, Swinburn BA. Reducing unhealthy weight gain in children through community capacity-building: results of a quasi-experimental intervention program, Be Active Eat Well. International Journal of Obesity. 2008;32:1060-7.

54. Sarrafzadegan N, Kelishadi R, Esmaillzadeh A, Mohammadifard N, Rabiei K, Roohafza H, et al. Do lifestyle interventions work in developing countries? findings from the Isfahan Healthy Heart Program in the Islamic Republic of Iran. Bulletin of the World Health Organisation. 2009:39-50.

55. Sautkina E, Goodwin D, Jones A, Ogilvie D, Petticrew M, White M, et al. Lost in translation? Theory, policy and practice in systems-based environmental approaches to obesity prevention in the Healthy Towns programme in England. Health & Place. 2014;29:60-6.

56. Schoen MW, Moreland-Russell S, Prewitt K, Carothers BJ. Social network analysis of public health programs to measure partnership. Social Science & Medicine. 2014;123:90-5.

57. Schuit AJ, Wendel-Vos GC, Verschuren WM, Ronckers ET, Ament A, Van Assema P, et al. Effect of 5-year community intervention Hartslag Limburg on cardiovascular risk factors. American Journal of Preventive Medicine. 2006;30:237-42.

58. Schulz AJ, Zenk S, Odoms-Young A, Hollis-Neely T, Nwankwo R, Lockett M, et al. Healthy Eating and Exercising to Reduce Diabetes: Exploring the Potential of Social Determinants of Health Frameworks Within the Context of Community-Based Participatory Diabetes Prevention. American Journal of Public Health. 2005;95:645-51.

59. Schwarte L, Samuels SE, Capitman J, Ruwe M, Boyle M, Flores G. The Central California Regional Obesity Prevention Program: Changing Nutrition and Physical Activity Environments in California's Heartland. American Journal of Public Health. 2010;100:2124-8.

60. Serpas S, Brandstein K, McKennett M, Hillidge S, Zive M, Nader PR. San Diego Healthy Weight Collaborative: A Systems Approach to Address Childhood Obesity. Journal of Health Care for the Poor and Underserved. 2013;24:80-96.

61. Simos J, Spanswick L, Palmer N, Christie D. The role of health impact assessment in Phase V of the Healthy Cities European Network. Health Promotion International. 2015;30:i71-i85.

62. Stafstrom M, Larsson S. The Trelleborg project: A process evaluation of a multi-sector community intervention to reduce alcohol consumption and related harm. Substance Use & Misuse. 2007;42:2041-51.

63. Stead M, Mackintosh AM, Eadie D, Hastings G. Preventing adolescent drug use: the development, design and implementation of the first year of 'NE Choices'. Drugs-Education Prevention and Policy. 2001;8:151-75.

64. Wagenaar AC, Gehan JP, Jones-Webb R, Toomey TL, Forster JL, Wolfson M, et al. Communities mobilizing for change on alcohol: Lessons and results from a 15-community randomized trial. Journal of Community Psychology. 1999;27:315-26.

65. Werna E, Harpham T. The implementation of the healthy cities project in developing countries: Lessons from Chittagong. Habitat International. 1996;20:221-8.
